# An Integrated Multi-omic Single-Cell Atlas of Human B Cell Identity

**DOI:** 10.1016/j.immuni.2020.06.013

**Published:** 2020-07-14

**Authors:** David R. Glass, Albert G. Tsai, John Paul Oliveria, Felix J. Hartmann, Samuel C. Kimmey, Ariel A. Calderon, Luciene Borges, Marla C. Glass, Lisa E. Wagar, Mark M. Davis, Sean C. Bendall

**Affiliations:** 1 Immunology Graduate Program, Stanford University, Stanford, CA, 94305, USA; 2 Department of Pathology, Stanford University, Stanford, CA, 94305, USA; 3 Department of Medicine, Division of Respirology, McMaster University, Hamilton, ON, L8S4K1, Canada; 4 Department of Developmental Biology, Stanford University, Stanford CA, 94305, USA; 5 Department of Surgery, Stanford University, Stanford CA, 94305, USA; 6 Department of Microbiology and Immunology, Stanford University, Stanford CA, 94305, USA

**Keywords:** B cells, human, mass cytometry, single cell, multi-omic, cell atlas

## Abstract

B cells are capable of a wide range of effector functions including antibody secretion, antigen presentation, cytokine production, and generation of immunological memory. A consistent strategy for classifying human B cells by using surface molecules is essential to harness this functional diversity for clinical translation. We developed a highly multiplexed screen to quantify the co-expression of 351 surface molecules on millions of human B cells. We identified differentially expressed molecules and aligned their variance with isotype usage, VDJ sequence, metabolic profile, biosynthesis activity, and signaling response. Based on these analyses, we propose a classification scheme to segregate B cells from four lymphoid tissues into twelve unique subsets, including a CD45RB^+^CD27^−^ early memory population, a class-switched CD39^+^ tonsil-resident population, and a CD19^hi^CD11c^+^ memory population that potently responds to immune activation. This classification framework and underlying datasets provide a resource for further investigations of human B cell identity and function.

## Highlights

• A mass cytometry screen reveals 98 surface molecules expressed by human B cells
• High-dimensional analysis identifies twelve B cell subsets across four tissues
• CD45RB, CD11c, CD39, CD73, and CD95 define subsets of antigen-experienced B cells
• Isotype usage, signaling, and metabolism vary in accordance with cell surface phenotype

## Graphical Abstract

**Figure:**
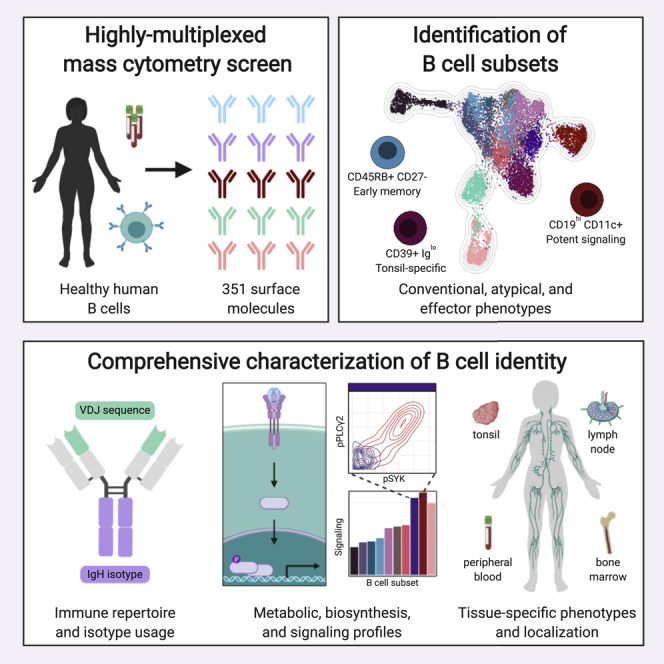

## Teaser

To identify molecules that distinguish functionally distinct subsets of human B cells, Glass et al. screened the expression of 351 surface molecules by mass cytometry. By combining identified molecules with functional readouts, they propose a new classification scheme to segregate B cells from four lymphoid tissues into twelve unique subsets.

## Introduction

B cells have the unique capacity to generate antibodies against a diversity of targets, providing protection against infection while also contributing to pathogenesis in settings of immune dysregulation. Antibody effector function is conferred through the immunoglobulin heavy chain (IgH) and is segregated into immature (IgM and IgD) and mature (IgG, IgA, and IgE) isotypes. Beyond antibody generation, B cells also contribute to immune responses through antigen presentation and cytokine production, which could be attributed, in part, to functionally specialized subsets (Cyster and Allen, 2019). Murine B cells are routinely classified on the basis of maturation status, antibody isotype, and effector function, but extrapolating to human B cell subsets has proven difficult because of both limitations in genetic tools and biological differences between species.

Canonical gating strategies segregate human B cells into five populations: transitional, naive, non-switched memory, switched memory, and plasma cells (Maecker et al., 2012), mostly capturing maturation, but not functional status. CD27 marks memory B cells, but even its earliest description indicated that there was IgH rearrangement in the CD27^−^ pool (Klein, Rajewsky and Küppers, 1998), and there are other reports of CD27^−^ memory phenotypes (Thorarinsdottir et al., 2016). IgH isotype is often used to augment B cell gating (Krishnamurty et al., 2016), but others have suggested that functional differences are better captured with phenotypic subsetting rather than isotypic subsetting (Zuccarino-Catania et al., 2014). These discrepancies highlight our inability to consistently identify and sort functional subsets of human B cells, impeding our capacity to selectively target pathogenic B cells in autoimmunity and induce memory responses in vaccination.

Our previous work provided a comprehensive analysis of B cell development in human bone marrow (Bendall et al., 2014). To characterize mature human B cells in the periphery, we developed a highly multiplexed single-cell screen to quantify the co-expression of 351 surface molecules by using mass cytometry. On the basis of our findings, we propose a classification scheme that subsets B cells from peripheral blood, bone marrow, lymph node, and tonsil into twelve unique populations and provide extensive single-cell profiles of cell surface phenotype, isotype usage, metabolism, biosynthesis activity, and signaling response to immune activation. This atlas of human B cell identity will enable further studies to interrogate functional B cell subsets in the context of homeostasis, vaccination, infection, autoimmunity, and cancer.

## Results

### A highly multiplexed single-cell surface screen reveals the human B cell surface proteome

**Figure 1:**
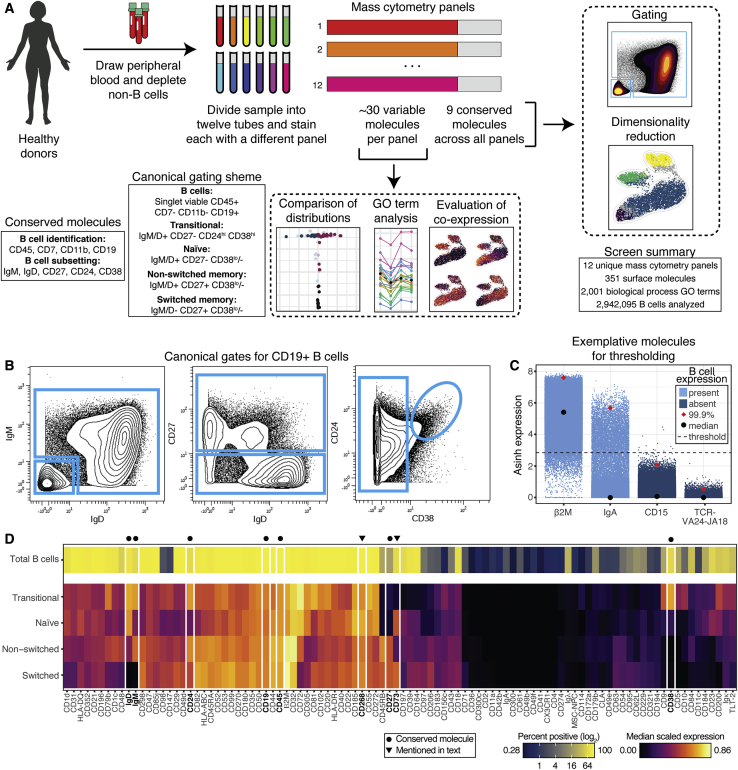
A highly multiplexed single-cell surface screen reveals the human B cell surface proteome (A) Experimental overview (n = 2 donors). (B) Representative gating of canonical populations. (C) Representative thresholding of positivity for molecules on the screen. (D) Percent positive of total B cells (top row) and median expression by subset (bottom rows) of molecules expressed by B cells.

To identify molecules that could potentially differentiate B cell subsets, we developed a highly multiplexed screen and quantified the co-expression of 351 surface antigens on healthy human B cells (n = 2 donors) (Figure 1A). We designed 12 mass cytometry antibody panels, each consisting of nine conserved molecules for subsetting, and ∼30 variable molecules that were unique to each panel, for discovery (Table S1). The conserved molecules were selected to facilitate gating into four canonical B cell subsets: transitional, naive, non-switched memory, and switched memory (Figures S1A and 1B). Plasma cells were left out of the analysis because of insufficient numbers after cryopreservation.

The collection of targets in the screen primarily consisted of surface molecules with immunology-associated gene ontology (GO) terms (Boyle et al., 2004), including hundreds of CD molecules (Figure S1B). After setting a stringent threshold for classifying a molecules as present or absent on donor-pooled B cells (Method Details; Figure 1C), we identified 98 surface molecules expressed on human B cells (Figure 1D). For each molecule, we quantified the percentage of positive B cells in the total sample and the median scaled expression value in the four canonical subsets. Expression patterns recapitulated known biology (e.g., near uniform expression of the B cell activating factor [BAFF] receptor, CD268), but also provided new insights. For example, the immunoregulatory ecto-nucleotidase, CD73, was enriched in naive and switched memory cells, but low to absent in transitional and non-switched memory cells. Interestingly, this molecule has been used to subset murine memory B cells (Tomayko et al., 2010), although its expression was mostly associated with non-switched memory cells in contrast to its expression in switched memory cells in our human dataset. Altogether, our single-cell screen facilitated robust identification of surface molecules expressed by human B cells.

### Differential expression analysis reveals the anergic profile of naive B cells

**Figure 2:**
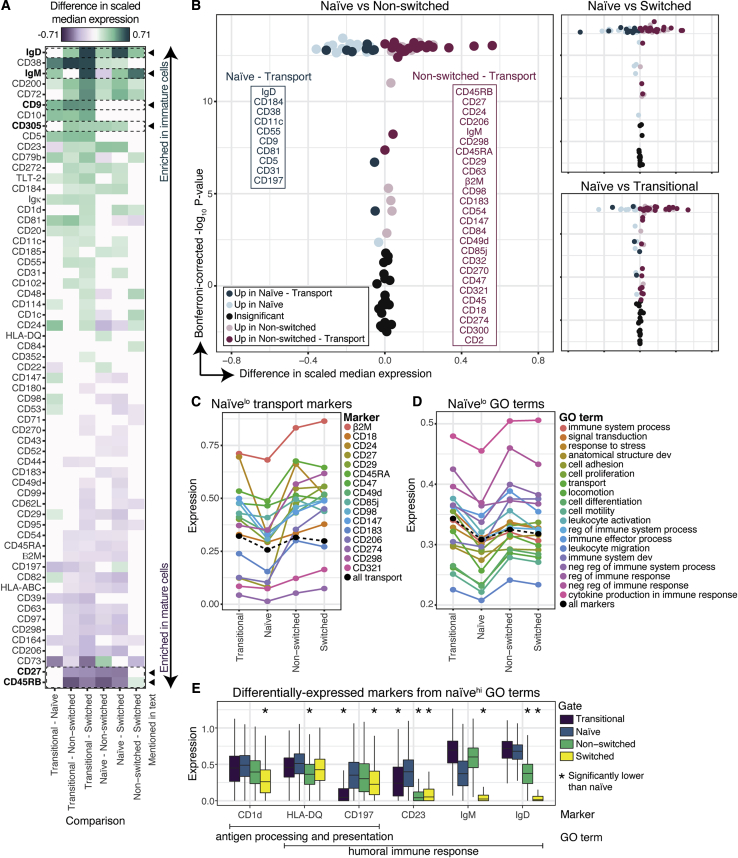
Differential expression analysis reveals the anergic profile of naive B cells (A) Difference in median expression for each pairwise comparison of subsets. All non-white tiles are significant (p < 0.005). (B) Volcano plots of the comparisons, colored by association with the GO term “transport.” Significantly different molecules listed in boxes are ordered by decreasing magnitude of difference of expression. (C) Median expression of transport molecules (colors). Mean of median expression of all transport molecules (black). (D) Mean of median expression of molecules associated with GO terms (color). Mean of median expression of all molecules (black). (E) Expression of six molecules more highly expressed in naive cells (p < 0.005).

The canonical gating scheme organizes B cells by their maturation status, from transitional through naive, non-switched, and switched memory. To interrogate the proteomic shifts that occur throughout this process, we evaluated differences in expression between each pairwise combination of donor-pooled subsets, for all molecules (Method Details). We plotted the difference in median expression for 61 differentially expressed molecules (p < 0.005, Kolmogorov-Smirnov (KS) test with Bonferroni correction) (Figure 2A).

As expected, the immature isotypes, IgD and IgM, were enriched in antigen-inexperienced cells (transitional and naive), whereas the canonical memory molecule, CD27, was enriched in memory cells. CD9, which has been reported to distinguish murine marginal zone, B-1, plasma (Won and Kearney, 2002), and/or regulatory (Sun et al., 2015) B cells, was enriched in transitional cells over all other subsets. CD305 (also known as leukocyte-associated Ig-like receptor 1 [LAIR-1]), which inhibits B cell receptor (BCR) signaling (van der Vuurst de Vries et al., 1999), was enriched in antigen-inexperienced cells over memory cells potentially increasing the antigen-specific activation threshold for these subsets. CD45RB (RB), an isoform of CD45, was preferentially expressed in memory cells, as has been reported in the tonsils (Jackson et al., 2009).

We then asked whether broader patterns in protein expression emerged from the pairwise comparison of B cell subsets. As an example, we plotted comparisons of naive cells to the other subsets and colored molecules associated with the GO term “transport” (Figure 2B), denoting molecules involved in trafficking across the cell membrane. We found naive cells expressed lower numbers of molecules associated with transport than any other subset, suggesting they are less responsive to stimuli. Indeed, naive cells had the lowest median expression value for 16 transport molecules and the lowest average expression across all 46 transport molecules (Figure 2C). We asked whether this trend was consistent across GO terms and found that naive cells had the lowest mean expression value for 19 out of 30 terms (Figure 2D). In fact, when the median expression values of all 98 molecules were averaged, naive cells had the lowest mean, suggesting they exist in a more anergic state than other B cell subsets.

Given that naive cells exhibited decreased expression of most molecules on the screen compared with that in other subsets, we then asked whether naive cells were enriched for any GO terms. Naive cells did not have the highest mean expression for any GO term and had the second highest mean expression for only two terms: “antigen processing and presentation” and “humoral immune response.” Within those two terms, only six molecules exhibited increased expression in naive cells over at least one other subset—CD1d, human leukocyte antigen (HLA)-DQ, CD197, CD23, IgM, and IgD (Figure 2E). In fact, in the entire screen, expression of only CD23, a non-classical Fc receptor that increases the threshold for B cell activation (Wang et al., 2015), was increased in naive cells over all other subsets. These findings confirm the anergic profile of naive B cells.

### CD45RB marks human memory B cells and identifies an early memory population

**Figure 3:**
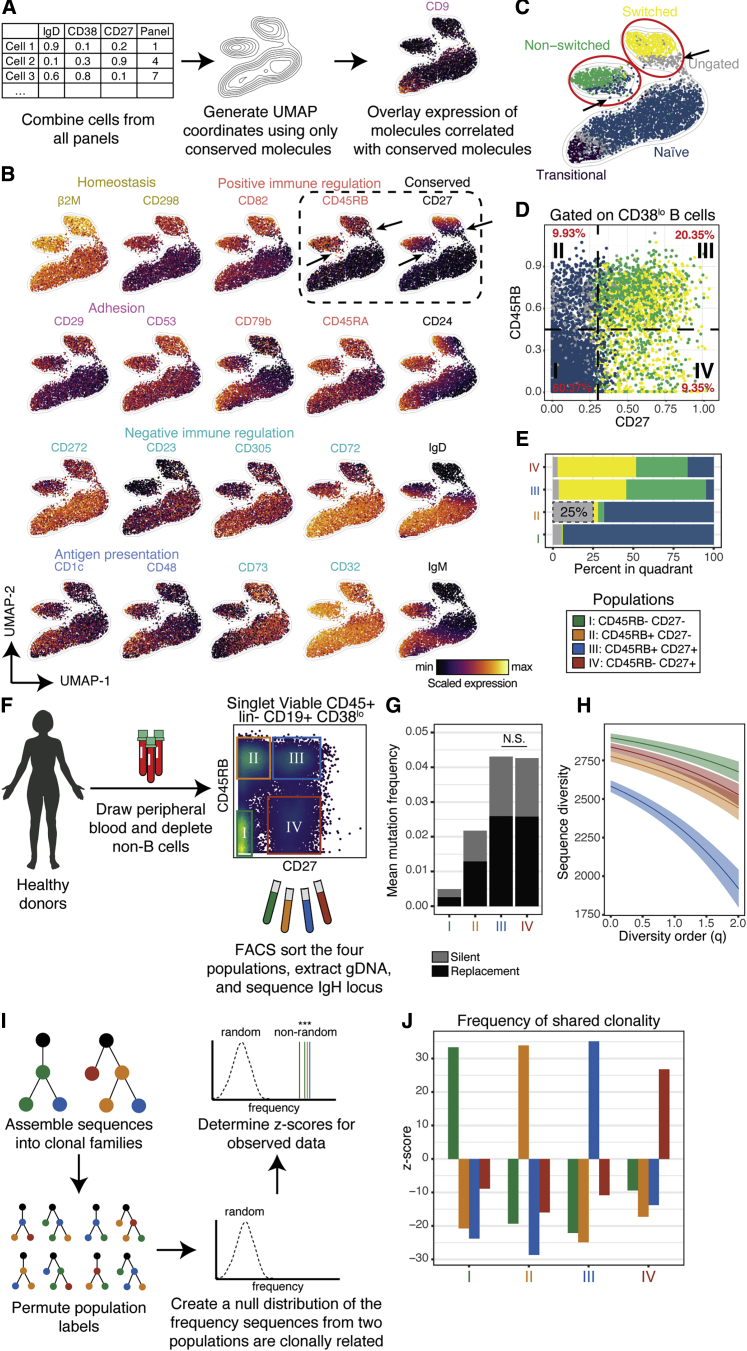
CD45RB marks human memory B cells and identifies an early memory population (A) Computational workflow. (B) UMAP plots of molecules correlated with the conserved molecule in their row. Arrows indicate RB^+^CD27^−^ cells. (C) UMAP plot colored by subset. (D) Biaxial plot colored by subset. Percent of CD38^lo^ B cells in each quadrant is quantified (red text). (E) Percent of cells in each quadrant from (D). (F) Experimental workflow. (G) Mean IgH mutation frequencies. All pairwise comparisons were significantly different (p < 0.005), except where indicated. (H) Sequence diversity across a range of diversity orders. Shaded regions indicate 95% confidence interval. (I) Computational workflow. (J) Z-scores of frequencies of shared clonality.

To find markers that uniquely identify distinct B cells not captured by canonical gating, we analyzed co-expression patterns of molecules across all B cells in an unbiased fashion. Because the surface screen was split across twelve tubes, we could not directly determine whether a molecule expressed on a cell in tube X was co-expressed with a molecule in tube Y. We therefore generated a Uniform Manifold Approximation and Projection (UMAP) plot (Becht et al., 2018) organized by the expression of the conserved molecules by using donor-pooled data from all twelve tubes (Method Details; Figure 3A). This two-dimensional representation of the high-dimensional data allowed us to visualize co-expression patterns of cells from different tubes in the same set of plots by overlaying molecule expression on UMAP coordinates.

Given that expression of the conserved molecules undergo coordinated changes throughout B cell maturation, we hypothesized that correlated molecules might also be useful for differentiating stages of B cell maturation. We plotted molecules that were correlated with the conserved molecules (Pearson method, ± r > 0.3) and organized them by function (label color) and by row for correlated conserved marker (Figure 3B). The highest correlation (r = 0.69) was between IgD and CD72, a negative regulator of B cell activation (Tsubata, 2012). IgD was also correlated with the negative regulators, CD23, CD305, and CD272 (Vendel et al., 2009). Although IgM was correlated with CD32, the inhibitory Fc receptor, it was inversely correlated with the immunoregulatory molecule CD73, which we found was enriched in naive and switched memory cells. CD27 was correlated with several molecules that positively regulate immune activation, potentially lowering the activation threshold of CD27^+^ cells.

Overlaying canonical gate labels on the UMAP coordinates revealed that two “islands” in the plot were not homogenously colored, indicating that although phenotypically similar, these cells were considered different subsets by canonical gating (Figure 3C). The switched memory island contained ungated cells, whereas the non-switched memory island contained both naive and ungated cells. Overlaying CD27 revealed that non-uniform expression of CD27 in these islands resulted in these mixed classifications. Overlaying RB, however, resulted in a more homogeneous coloring of the two memory populations while retaining an absence of expression in the antigen-inexperienced island. The majority of CD27^+^ cells were also RB^+^, whereas the RB^+^CD27^−^ population contained 25% ungated cells (Figures 3D and 3E). Given the co-localization of RB^+^CD27^−^ cells and CD27^+^ cells on the UMAP, we hypothesized that that these cells represent a population of memory cells that was not recognized under the current classification scheme.

To assess the spectrum of memory cell specification across the RB and CD27 compartments, we prospectively isolated the four quadrants of the CD27 × RB biaxial from healthy, human B cells (n = 2 donors) and sequenced the IgH loci by next-generation sequencing (NGS) (Method Details; Figure 3F). As a proxy for antigen exposure, we measured the donor-pooled mutation frequency of nucleotides in the IgH loci outside of the complementarity-determining region 3 (CDR3) (Figure 3G) (Boyd and Crowe, 2016). As expected, CD27^+^ cells had a relatively high mutational burden, acquired through somatic hypermutation (SHM) after exposure to antigen. Conversely, RB^−^CD27^−^ cells showed a low mutational burden given that they are still naive to antigen (p < 0.005, Wilcoxon rank sum test with Bonferroni correction). Interestingly, RB^+^CD27^−^ cells displayed an intermediate mutational burden, higher than RB^−^CD27^−^ cells and lower than CD27^+^ cells, indicating that they have been exposed to antigen, but undergone fewer cycles of SHM than other memory cells.

During an immune challenge, B cells that are reactive to relevant antigens are selected to proliferate and differentiate into effector and memory classes. Naive cells, therefore, tend to have more diverse immune repertoires than memory cells because they have not undergone selection (Briney et al., 2012). We quantified the diversity of the four populations across a range of diversity orders (Hafler et al., 2014) and found that RB^−^CD27^−^ cells had the highest diversity, whereas RB^+^CD27^+^ cells had the lowest diversity (Method Details; Figure 3H). Both RB^+^CD27^−^ cells and RB^−^CD27^+^ cells had intermediate levels of diversity, suggesting RB^+^CD27^−^ cells undergo less selection than RB^+^CD27^+^ cells. This might also be true for RB^−^CD27^+^ cells, but given their high mutational burden, it might instead be indicative of a long-lived memory population that mediates protection against a lifetime of past immune challenges.

If expression of RB was random and irrelevant to B cell activation and maturation, we would expect RB^+^ and RB^−^ cells to share clonal lineages, given that the molecule would not meaningfully segregate cells. We therefore asked whether cells from one population tend to be clonally related to cells from any other population (see Method Details; Figure 3I). We found that cells from each of the four populations were much more likely to share clonal lineages with cells from the same population than with those from a different population (Figure 3J). This indicates that expression of RB and CD27 are highly coordinated within clonal lineages, as would be expected of two molecules that are expressed in response to antigen engagement. Altogether, these findings provide strong evidence that expression of RB is indicative of a peripheral blood memory B cell and, in conjunction with an absence of CD27, can be used to classify an early memory population.

### Segregating B cells into phenotypically and isotypically distinct subsets

**Figure 4:**
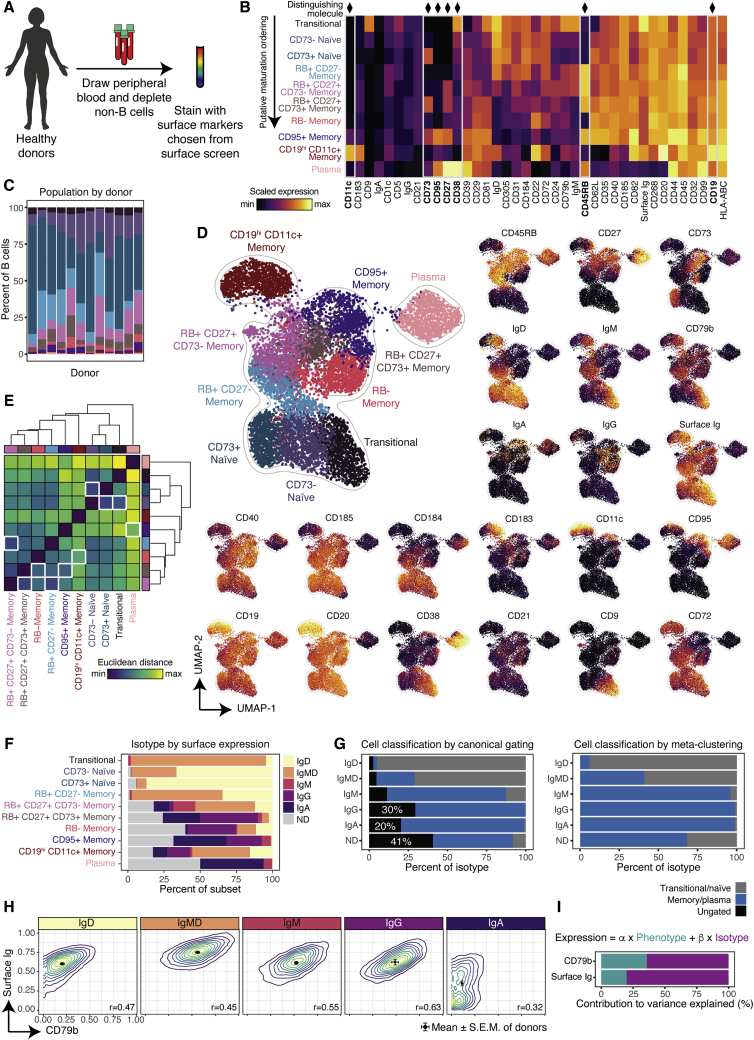
Segregating B cells into phenotypically and isotypically distinct subsets (A) Experimental workflow (n = 3 donors). (B) Median expression by subset. (C) Percent of B cells for all subsets, colored as in (B). (D) UMAP plot generated from an equal subsampling by subset. (E) IgH isotype usage by subset. ND denotes “not determined”; IgMD denotes co-expression of IgM and IgD. (F) Euclidean distance between each B cell subset based on median expression profile. White boxes denote column minimum. (G) Subset composition by isotype as determined by canonical gating or meta-clustering. (H) Contour plots by IgH isotype. Dots and error bars indicate mean and SEM of individual donors. (I) Relative contribution of phenotype and isotype to the variance explained by linear models created to predict single-cell expression of CD79b or surface Ig.

The surface screen revealed dozens of molecules differentially expressed in B cells and resulted in the identification of a memory population, so we hypothesized that we could classify B cells into more granular subsets. We stained fresh, healthy, human peripheral blood B cells (n = 3 donors) with a mass cytometry panel comprised of the most informative molecules from the screen (Figure 4A), including canonical B cell molecules, heavy and light chain isotypes, and molecules with multi-modal distributions (Table S1).

Donor-pooled cells were clustered into ten distinct populations, including two naive and six memory subsets (Method Details; Figure 4B). Their surface expression profiles were plotted and arranged in a putative maturation order on the basis of IgH isotype usage and expression of maturation molecules (Figure 4B, top to bottom). The characteristic expression pattern of seven distinguishing molecules were sufficient to manually gate each population and thus were also used to label the populations in this scheme: CD11c, CD73, CD95, CD27, CD38, RB, and CD19 (Figure S2B). There was some variation in subset size between donors, but all donors contained cells from all ten subsets across datasets assessed in this report (n = 12) (Figure 4C).

Each subset was equally subsampled from donor-pooled cells, plotted by UMAP, and colored by either subset or molecule expression (Figures 4D and S3). Subsets tended to form unique islands on the plot, providing an orthogonal validation of our classification methodology. Transitional cells co-localized with CD73^−^ and CD73^+^ naive cells in a single island, forming a gradient of diminishing IgM and IgD, despite the UMAP plot being generated by using only phenotypic, not isotypic molecules (Figure 4D). Although there was a clear separation between antigen-inexperienced and memory subsets (notably bridged by the RB^+^CD27^−^ memory population), no single molecule was sufficient to discriminate memory from antigen-inexperienced cells. Plasma cells were largely RB^+^ and mostly lacked surface Ig as assessed by surface light chain expression (Method Details; Figure S2C). Some IgA^+^ and IgM^+^ plasma cells retained surface expression of Ig as previously reported (Pinto et al., 2013), but we did not observe expression of surface IgG or IgD on plasma cells.

Three of the six memory populations organized into a distinct island: RB^+^CD27^−^ memory (which are also CD73^−^), RB^+^CD27^+^CD73^−^ memory, and RB^+^CD27^+^CD73^+^ memory. Much like in the transitional to naive gradient, differential isotype usage can be seen in these memory subsets with RB^+^CD27^−^ memory favoring IgD, RB^+^CD27^+^CD73^−^ favoring IgM, and RB^+^CD27^+^CD73^+^ favoring IgG and IgA. These data could be indicative of a maturation continuum that occurs after antigen exposure, though these populations might arise concomitantly. RB^−^ memory was mostly class-switched, but had a more heterogenous phenotypic profile, which complemented its high clonal diversity, with varying levels of CD27, CD73, and CD183 (also known as CXCR3), a chemokine receptor that facilitates homing to inflamed tissue (Kaminski et al., 2012). CD95^+^ memory, uniquely defined by expression of the death receptor, CD95 (also known as FasR), also had heterogenous expression of several molecules, including CD11c and CD73, but tended to be class-switched, RB^+^, CD27^+^, and CD72^−^. We observed that several memory populations could also be further subdivided but were not further analyzed in this report: CD5^+^CD1c^+^IgMD^+^RB^+^CD27^−^CD73^−^CD38^+^ cells and CD9^+^CD22^−^IgA^+^RB^+^CD27^+^CD73^−^ cells.

Of particular note were CD19^hi^CD11c^+^ memory, which have overlapping features with previously described T-bet^+^ B cells (Karnell et al., 2017). This subset formed a unique island characterized as CD21^−^, CD20^hi^, CD38^−^, CD73^−^, CD40^lo^, and RB^−^. Notably, this population largely lacked expression of the chemokine receptors CD185 (also known as CXCR5) and CD184 (also known as CXCR4), suggesting these cells might not participate in germinal center (GC) responses or might be recent GC emigrants (Cyster and Allen, 2019). These cells could be further delineated on the basis of isotype usage, with IgMD^+^ cells tending to be CD27^+/lo^, CD185^lo^, CD183^+^, and CD95^+/−^ and class-switched cells tending to be CD27^−/lo^, CD185^−^, CD183^−^, and CD95^−^. To further investigate this heterogeneity, we analyzed a public dataset that contained mass cytometry data of healthy donors by using a general immunophenotyping panel (n = 5 donors) and a B-cell-centric panel (n = 3 donors, Hartmann et al., 2019). We confirmed that the CD19^hi^ CD11c^+^ memory subset was significantly enriched for T-bet positivity over other B cells (medians: 51% and 4% positive for T-bet, p < 0.005, Wilcoxon signed rank test) (Figures S4A and S4B). Within CD19^hi^CD11c^+^ memory, T-bet and CD27 were negatively correlated (r = −0.55, Pearson method), so we hierarchically clustered cells on the basis of expression of these markers, generating a CD27^−^ T-bet^+^ population and CD27^+/−^ T-bet^−^ population (Figure S4C). We found an enrichment for IgG usage in T-bet^+^ cells (Figure S4D), and diminished expression of CD21, CD40, CD45, and CD45RA (p < 0.005) (Figure S4E). Notably, T-bet^+^ cells were enriched for programmed cell death protein 1 (PD-1) (Figures S4E and S4F). Because these cells are present in higher proportion in various autoimmune conditions than in healthy controls (Karnell et al., 2017), they might contribute to the adverse autoimmune events that are associated with anti-PD-1 therapy (Abdel-Wahab, Shah and Suarez-Almazor, 2016).

To assess phenotypic similarity between subsets, we calculated the pairwise Euclidean distance between median expression profiles (Figure 4E). For each population, we quantified the subset that was most phenotypically similar (Figure 4E, white boxes). RB^+^CD27^−^ memory and RB^+^CD27^+^CD73^−^ were most similar to each other, further validating the status of RB^+^CD27^−^ cells as a memory subset. Interestingly, plasma cells were most similar to CD95^+^ memory, mirroring their proximity on the UMAP (Figure 4D), whereas CD95^+^ memory was most similar to RB^+^CD27^+^CD73^+^ memory, another population enriched for class-switched isotypes.

IgH isotype was not used to meta-cluster cells, but organizing B cells by phenotype resulted in an organization by isotype as well, in pooled data (Figure 4F) and across individual donors (Figure S2D). Importantly, less than 0.26% of any transitional/naive subset expressed a mature isotype. As class-switch recombination occurs only after activation, naive cells are, by definition, not class-switched (Cyster and Allen, 2019). By canonical gating, 30% of IgG^+^ cells and 20% of IgA^+^ cells were left ungated because of an absence of CD27, demonstrating the insufficiency of CD27 alone as a memory molecule (Figures 4G and S2E). In contrast, our approach correctly classified more than 99% of IgG^+^ and 98% of IgA^+^ cells as memory cells. Furthermore, by canonical gating, 45% of cells with indeterminate isotype were left unannotated. These cells are not mislabeled IgE^+^ B cells as they are exceedingly rare in healthy blood (Jiménez-Saiz et al., 2019) (Figure S2G). This is an important consideration in cytometry panel design as IgMD^−^ is often used as a proxy for IgG^+^ or IgA^+^. Ig^lo/−^ cells, which comprised 10% of total B cells (Figure S2F), were found across all phenotypes, and likely encompass a mixture of all isotypes. It is therefore essential to include probes against all four major isotypes if comparisons are to be made.

Ig isotype usage is known to affect downstream effector function and differentiation patterning (Dogan et al., 2009). As such, we also organized B cells on the basis of isotype and observed distinct patterns of expression of two components of the BCR complex: surface Ig and CD79b (Figure 4H). Surprisingly, the mature isotype, IgA, had the lowest expression numbers of both molecules despite the increased sensitivity of antigen-experienced B cells (Kurosaki, Kometani and Ise, 2015). We also found that although CD79b and surface Ig expression were correlated in each isotype, IgG^+^ B cells had the highest correlation between surface Ig and CD79b expression (r = 0.63, Pearson method), whereas IgA^+^ B cells had the lowest (r = 0.32), suggesting IgA^+^ cells do not solely rely on CD79 for signaling and might instead possess unique regulatory framework downstream of the BCR.

Given these trends, we asked whether phenotype or isotype contributed more to predicting expression amounts of surface Ig and CD79b. We created single-cell multiple linear regression models in which a cell’s phenotypic label (e.g., RB^+^CD27^−^ memory) and isotypic label (e.g., IgA^+^) were used to predict the expression of either CD79b or surface Ig (Method Details; Figure 4H). Although both were informative, a cell’s isotype contributed more than a cell’s phenotype for predicting the expression of the two molecules. Altogether these findings demonstrate that our high-dimensional classification organizes peripheral blood B cells into ten phenotypically distinct subsets with a more accurate partitioning of cells than canonical gating strategies. Additionally, these phenotypic partitions displayed isotypic restriction that further contributed to a B cell’s identity.

### Interrogation of B cell subset function reveals differential metabolic, biosynthesis, and immune signaling activity

**Figure 5:**
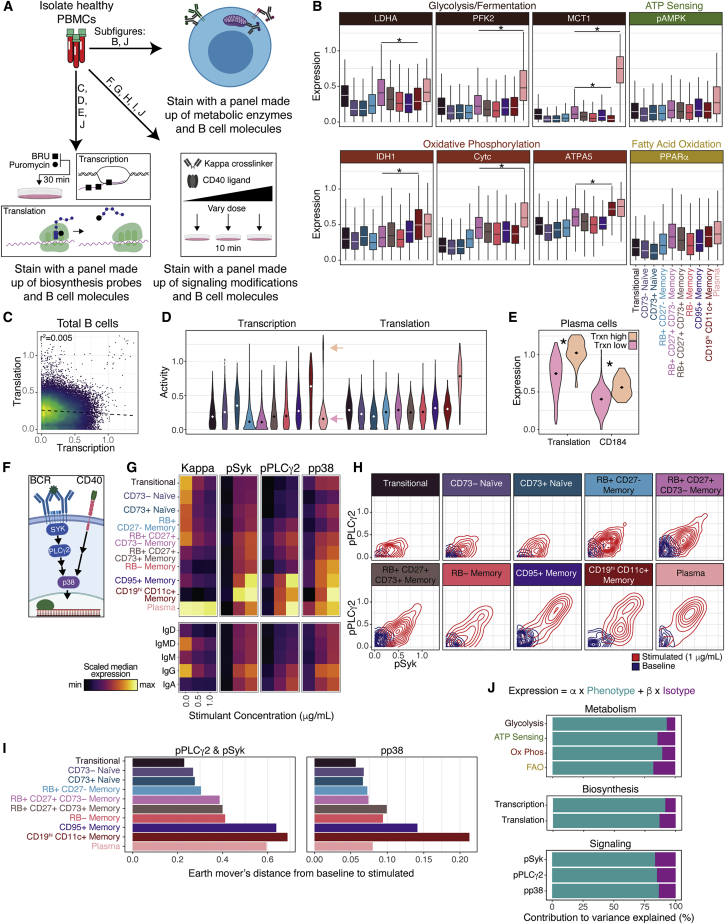
Interrogation of B cell subset function reveals differential metabolic, biosynthesis, and immune signaling activity (A) Experimental workflow (n = 9 donors). (B) Expression of metabolic enzymes. Stars indicate significance (p < 0.005). Boxes represent interquartile range (IQR) and whiskers represent IQR +/− 1.5*IQR (C) Biaxial of biosynthesis activity. (D) Violin plots of biosynthesis activity. Bimodality indicated by arrows. (E) Violin plots of significantly differentially expressed molecules (p < 0.005). (F) Signaling diagram. (G) Median expression of Igλ^−^ B cells, grouped by phenotype or isotype. (H) Contour plots of signaling molecules. (I) Quantification of earth mover’s distance from baseline samples to stimulated samples (1 μg/mL) for pPLCγ2 and pSyk and pp38. (J) Relative contribution of phenotype and isotype to the variance explained by linear models created to predict single-cell expression of metabolic pathways, biosynthesis activity, or cell signaling.

To investigate the functional properties of our refined B cell classification scheme, we asked whether surface profile denoted differences in other underlying functional cell processes. We stained healthy, human peripheral blood mononuclear cells (PBMCs) from additional donors (n = 9 donors) with mass cytometry panels to interrogate B cell metabolic profiles, biosynthesis activity, and immune signaling (Figure 5A; Table S1).

To assess single-cell metabolic profiles (Hartmann et al., 2020), we quantified the expression of eight enzymes, associated with four metabolic pathways: glycolysis or fermentation, ATP sensing, oxidative phosphorylation (ox-phos), and fatty acid oxidation (Figure 5B). All subsets expressed all enzymes, but expression levels varied by phenotype. Naive cells had the lowest expression of all subsets, which synergizes with the anergy observed in their surface proteomes, whereas RB^+^CD27^−^ memory cells had an intermediate metabolic profile between naive and memory subsets. Plasma cells had the highest median expression for all enzymes and were significantly higher than other B cell subsets for molecules associated with both ox-phos and glycolysis (p < 0.005). Outside of plasma cells, RB^+^CD27^+^CD73^−^ memory and CD19^hi^CD11c^+^ memory cells had the highest median expression for all enzymes. Interestingly, CD19^hi^CD11c^+^ memory was higher than RB^+^CD27^+^CD73^−^ memory for two ox-phos enzymes, but lower for the glycolytic enzyme MCT1 (p < 0.005). These differences in pathway usage might be because of different functional roles and therefore different metabolic needs.

We recently developed an assay to quantify *de novo* RNA and protein synthesis in parallel with functional and phenotypic characteristics by combining 5-Bromouridine (BRU) and puromycin labeling with mass cytometry (Kimmey et al., 2019). Applying this approach to healthy human B cells, we found transcriptional activity explained very little of the variance observed in translational activity (r^2^ = 0.005) (Figure 5C), highlighting the differential regulation of these two processes. CD19^hi^CD11c^+^ memory cells had the highest median transcriptional activity, followed by CD73^+^ naive cells, which had the lowest median translational activity (Figure 5D). Given the anergy observed in the naive department, it was surprising to see such a high level of transcriptional activity in these cells, and it is unclear what transcripts are being synthesized given the low translational activity in these cells. Plasma cells had the highest median translational activity but displayed bimodal transcriptional activity. We asked whether any other molecules were differentially expressed between transcription^hi^ and transcription^lo^ plasma cells and found that translational activity and CD184 expression were higher in transcriptionally active plasma cells than in transcription^lo^ plasma cells (p < 0.005) (Figure 5E). This transcriptionally active population might be long-lived plasma cells whereas the transcriptionally inactive population might be short-lived plasma cells. Long-lived plasma cells have been observed to increase expression of CD184 to facilitate bone marrow homing and would require continuous transcriptional activity to facilitate constitutive Ig production and secretion (Nutt et al., 2015).

Given that transcriptional and translational activity were uncorrelated in total B cells, but positively correlated in plasma cells, we asked whether the relationship between transcriptional and translational activity varied by phenotype. We fit simple linear models to interrogate the relationship between transcriptional activity and translational activity in transitional/naive clusters and separately in memory clusters (Figure S3A). Transcriptional and translational activity in transitional/naive clusters had a strongly negative relationship (r^2^ = 0.61, p < 1^−10^), but were uncorrelated in memory cells (r^2^ = 0.02, p = 1.32). Total Ig amounts (measured by intracellular staining) correlated with translational activity in both transitional/naive (r^2^ = 0.66, p < 1^−12^) and memory (r^2^ = 0.28, p < 1^−3^) clusters, but each regression had different coefficients and intercepts, so total Ig was only predictive of translational activity if the phenotypic subset was considered, highlighting the importance of proper subsetting in discovery and interpretation of biological findings.

To assess differences in immune activation sensitivity between subsets, we stimulated B cells with varying doses of BCR crosslinker (anti-kappa light chain) and CD40 ligand (CD40L) for 10 min and fixed and stained them with a mass cytometry panel that included antibodies against phosphorylated targets intrinsic to B cell signaling (Figure 5A; Table S1). We measured phosphorylation of spleen tyrosine kinase (pSYK) and the downstream phospholipase Cγ2 (pPLCγ2), two molecules involved in the signaling cascade caused by antigen recognition mediated by the BCR complex (Figure 5F) (Kurosaki, Shinohara and Baba, 2010). We also measured phosphorylation of the stress-activated protein kinase p38 (pp38), which is strongly induced by CD40 stimulation, a molecule activated during antigen presentation to T cells and weakly induced by BCR stimulation (Sutherland et al., 1996). Phosphorylation of p38 can also be induced by Toll-like receptor (TLR) stimulation (Kawai and Akira, 2006), but response to TLR ligands was not evaluated in this study.

We segregated donor-pooled Igλ^−^ B cells by subset and isotype and assessed the median levels for each regulatory phosphorylation as a function of stimulant dose (Figure 5G). As expected, total kappa light chain diminished after stimulation as surface Ig was crosslinked, internalized, and degraded. Unsurprisingly, IgM^+^ and IgD^+^ cells had lower levels of signaling in response to stimulation, whereas cells with the mature isotypes, IgG and IgA, were the most potent responders. As we had seen in our previous datasets, IgA^+^ cells had the smallest quantity of Ig at baseline, yet responded with comparable potency to IgG^+^ cells, which had the highest quantity of Ig at baseline. This is particularly surprising as we had previously observed that IgA^+^ cells also had the lowest expression of the BCR signaling molecule, CD79b. Segregating class-switched cells by phenotype, we found little difference in signaling response between IgG^+^ and IgA^+^ cells of the same phenotype, despite their differences in BCR expression (Figure S3B). The only exception was plasma cells, in which IgG^+^ cells had weak responses compared with those of IgA^+^, presumably because of a lack of surface Ig on IgG^+^ cells. These findings highlight that signaling potency is poorly explained by BCR copy number.

We visualized changes in phosphorylation state of the two molecules in the BCR complex signaling cascade, SYK and PLCγ2, on biaxial contour plots and found stark contrasts in distribution shifts between subsets (Figure 5H). Although all populations responded to stimulation, only the plasma and memory subsets (particularly CD95^+^ memory and CD19^hi^CD11c^+^ memory) contained a highly responsive, double-positive population. To quantify the signaling response, we calculated the earth mover’s distance between baseline and stimulated cells and found that these two memory populations, along with plasma cells, were notably more responsive than all other subsets (Figures 5I, left, and S3C). Interestingly, the other seven populations self-organized from least to most responsive when arranged by putative ordering of maturation (Figure 4), suggesting that sensitivity to BCR-specific activation increases with maturation.

To assess CD40 signaling, we also quantified the distance between baseline and stimulated cells for pp38, (Figures 5I, right, and S3D). Although some of the trends were similar to the BCR complex signaling molecules (e.g., antigen-inexperienced cells were less responsive than memory cells), some differences emerged. Plasma cells had much lower signaling than in the BCR complex pathway, which is unsurprising because their primary function is antibody production, not T cell stimulation (Cyster and Allen, 2019). CD19^hi^CD11c^+^ memory signaling was uniquely high, despite having the lowest baseline expression of CD40, other than plasma cells (Figure 4B).

Given the heterogeneity of functional activity we observed across B cells, we asked whether this variance was best explained by our phenotypic labels or by isotype. Using the same multiple linear regression approach used to quantify contributions to surface Ig and CD79b (Figure 4H), we quantified the relative contribution of phenotype and isotype usage to predict expression of metabolic pathway expression, biosynthesis activity, and signaling response (Figure 5J). In contrast to surface Ig and CD79b expression, a cell’s phenotype was much more informative than a cell’s isotype for predicting its metabolic, biosynthesis, and signaling profiles. Collectively, these findings demonstrate that our phenotypic classification captures functional distinctions in metabolic pathway usage, biosynthetic activity, and signal response to immune activation.

### Characterization of lymphoid-tissue-specific B cell populations

**Figure 6:**
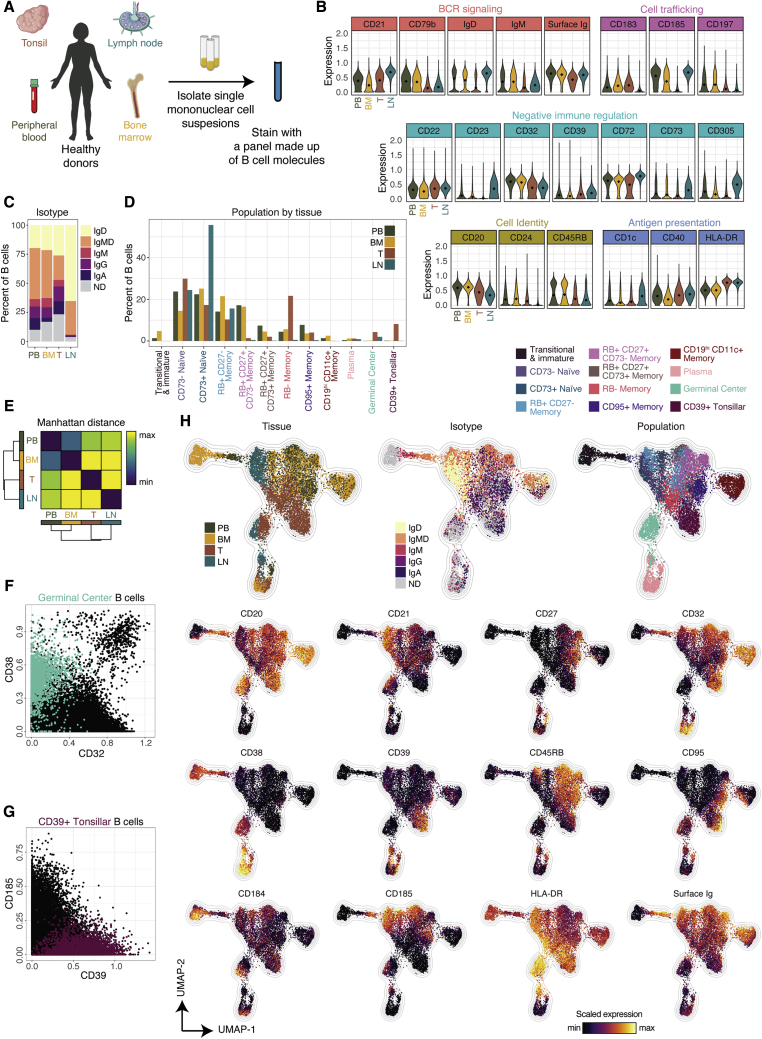
Characterization of lymphoid tissue-specific B cell populations (A) Experimental workflow (n = 11 donors). (B) Violin plots of molecules significantly differentially expressed by at least two tissues (p < 0.005). (C) IgH isotype usage by tissue. ND denotes “not determined”; IgMD denotes co-expression of IgM and IgD. (D) Subset composition by tissue. (E) Manhattan distance between each tissue based on subset composition. (F) Biaxial of B cells colored as GC or other. (G) Biaxial of B cells colored as CD39^+^ tonsillar or other. (H) UMAP plot generated from an equal subsampling by tissue, then an equal subsampling by subset.

To broaden the scope of our human B cell profiling beyond peripheral blood, we profiled bone marrow (n = 3), tonsil (n = 3), lymph node (n = 1), and additional peripheral blood samples (n = 4) from a new cohort of healthy donors (n = 11) by mass cytometry (Figure 6A; Table S1). As expected, tonsil and lymph node were heavily enriched for B cells, as compared to those in peripheral blood and bone marrow (Figure S6A).

To interrogate global differences in B cell expression between tissues, we evaluated differences in expression between each pairwise combination of donor-pooled tissues, for all molecules. We identified 21 molecules that were differentially expressed (p < 0.005) between at least one pair of tissues and plotted their distributions, organized by function (Figure 6B). HLA-DR, which facilitates antigen presentation to T cells, was expressed at high amounts in tonsil and lymph node, two tissues that promote T:B interactions. CD32, an inhibitory Fc receptor, had low expression in tonsil and lymph node, as might be expected in a microenvironment promoting B cell activation. Adversely, several inhibitory molecules were enriched in lymph node, including CD23, CD72, CD73, and CD305. The lymph node also had substantial skewing toward immature isotypes (Figures 6C and S6B). Tonsil, however, was not enriched for any inhibitory molecule (Figure 6B) and had the largest proportion of IgG^+^, IgA^+^, and undetermined isotypes, which are primarily composed of cells with memory phenotypes (Figures 6C and 4G).

To evaluate the composition of B cell phenotypes within tissues, we plotted subset proportions and, in accordance with the isotype data, we found that the lymph node was heavily enriched for CD73^+^ naive cells (> 50%) (Figures 6D and S6C). In fact, all memory populations in the lymph node were nearly absent (< 0.5%) save for RB^+^CD27^−^ memory. The tonsil, however, did not exhibit this same skewing toward naive cells; it had greatly diminished proportions of RB^+^CD27^+^CD73^−^ memory and RB^+^CD27^+^CD73^+^ memory, but an enrichment of RB^−^ memory (> 20%). CD19^hi^CD11c^+^ memory was absent in both the tonsil and lymph node. Transitional cells were also absent in both tonsil and lymph node and enriched in bone marrow, though the definition also encompassed immature B cells, which were present exclusively in the bone marrow. Plasma cells were present at the lowest amounts in the blood, and this phenomenon might be underrepresented here because of cell loss from cryopreservation. To evaluate the dissimilarity of tissues, we calculated the pairwise Manhattan distance between each tissue on the basis of subset composition (Figures 6E and S6D). Each lymphoid tissue was most similar to peripheral blood, and peripheral blood was most similar to bone marrow.

We identified two subsets absent in peripheral blood: germinal center (GC) B cells, present in both the tonsil and lymph node, and a CD39^+^ tonsillar population, present exclusively in the tonsil as ∼8% of total B cells (Figures 6D and S6E). GC cells were CD38^+^ and CD32^−^ (Figure 6F) as previously described (Macardle et al., 2002), whereas CD39^+^ tonsillar cells were CD185^−^ (Figure 6G). CD39 is an ectoenzyme that regulates immune responses by converting ATP into AMP, which can be dephosphorylated into adenosine by CD73 (Antonioli et al., 2013). To our knowledge, this population has not previously been reported and might represent a tissue-resident precursor to other memory and effector phenotypes.

To better characterize the heterogeneity present in our lymphoid tissues, tissue- and subset-subsampled cells were plotted by UMAP (Method Details; Figures 6H and S7). In parallel, we assessed differential expression between tissues within subsets, as well as tissue-specific subset expression profiles (Figures S6E and S6F). Cells derived from the tonsil and lymph node largely occupied unique areas of the UMAP, whereas bone marrow and peripheral blood tended to overlap, as expected from our quantification of dissimilarity (Figure 6E). Compared with RB^+^CD27^−^ memory in peripheral blood, RB^+^CD27^−^ memory in the lymph node was particularly distinct with differential expression of 18 molecules, including higher expression of the chemokine receptors CD184 and CD185, the antigen-presentation-related molecules CD1c and CD40, and the negative immune regulators CD23, CD39, and CD72 (Figure S6E).

GC B cells formed a distinct island on the UMAP, nested between memory and plasma cells, as would be expected given that germinal centers give rise to both plasma and memory cells (Cyster and Allen, 2019). In fact, several GC cells localized with plasma and RB^−^ memory cells, which might reflect cells actively transitioning into these phenotypes. GC cells have low surface Ig amounts, comparable to plasma cells, making isotype distinction difficult. It is, however, clear from those cells with sufficient expression to classify that although perhaps enriched for mature isotypes, the population contains IgD and IgMD cells, despite GC cells being canonically defined as IgD^−^ (Victora and Nussenzweig, 2012). These cells are distinct from other populations described here because they are RB^−^CD27^−^CD24^−^CD39^−^CD73^−^ and CD32^−^, in addition to being CD38^+^ and CD95^+/−^ (Figure S7).

GC B cells can be further subdivided by their spatial location within a germinal center—dark zone (DZ) B cells undergo division and somatic hypermutation, whereas light zone (LZ) B cells undergo selection through interactions with follicular dendritic cells and T cells (Victora and Nussenzweig, 2012). CD184 has been reported as enriched in DZ GC B cells and CD185 in LZ GC B cells (Victora et al., 2012), but in our dataset the markers were positively, not negatively correlated, as would have been expected given their proposed opposing roles in trafficking. The two markers instead mostly captured tissue-specific differences because double positives were enriched in lymph node and double negatives were enriched in tonsil (Figure S6G).

Tonsillar CD39^+^ B cells also formed a distinct area on the UMAP, situated next to RB^−^ memory and CD95^+^ memory (Figure 6H). These cells were also characterized by low expression of surface Ig and were enriched for class-switched isotypes, particularly IgA. They were CD32^+^, had mixed expression of RB, CD27, CD24, CD11c, CD95, and CD183, low to no expression of CD21, CD72, and CD73, and completely lacked expression of CD38 (Figure S7). Those tonsillar CD39^+^ cells that were CD11c^+^ also tended to be CD19^hi^, but were distinguishable from CD19^hi^CD11c^+^ memory (which were absent in the tonsil) by their lower expression of CD20, CD22, and CD72, and higher expression of CD39, CD40, and CD184. It is possible, however, that CD39^+^ tonsillar cells are a precursor to CD19^hi^CD11c^+^ memory.

Plasma cell profiles also varied by tissue (Figure S6F). Whereas peripheral blood plasma cells were characterized as CD38^+^CD27^hi^, plasma cells from all other tissues were CD38^hi^CD27^+/−^. High expression of HLA-DR, mixed expression of CD95, and an absence of CD32 uniquely distinguished lymph node plasma cells (Figure 6H). Bone marrow and tonsil plasma cells were most similar (Figure S6F), unified by high expression of CD32, an enrichment of CD184, mixed expression of CD183, and low to absent expression of HLA-DR and RB (Figure S7). As evaluted by surface expression, peripheral blood and tonsil plasma cells were enriched for IgA, whereas lymph node and bone marrow cells were enriched for IgM.

Altogether, these data reveal the tissue-specific profiles and proportions of the ten subsets identified and introduce extensive phenotypic profiles of GC B cells and CD39^+^ tonsillar B cells. We report a gating scheme for tonsil (also applicable to lymph node) to capture all populations present in these tissues by using only six markers: CD27, CD38, CD39, RB, CD73, and CD95 (Figure S6H). These findings, in combination with our evaluation of metabolic pathway usage, biosynthetic activity, and signal response to immune activation, provide comprehensive descriptions of twelve distinct B cell subsets (Figure S8). These subset definitions represent a framework to understand and assess the functional contributions of B cells to human immunity.

## Discussion

To interrogate deep phenotypic diversity in primary cells, we developed a highly multiplexed single-cell surface screen and applied it to identify molecules that could segregate subsets of human B cells. This approach enabled us to differentiate twelve B cell subsets across four lymphoid tissues and relate their functional profiles. Importantly, these populations could also be manually gated for prospective isolation by fluorescence-activated cell sorting (FACS), though purity and yield might suffer without high-dimensional profiles. These subsets built and expanded upon an existing understanding of B cell identity, including the utility of CD27 as a memory marker, IgH isotype restriction within subsets, and coarse definitions of transitional, naive, memory, and plasma cells. We found no obvious innate-like B1 B cell phenotype (Cyster and Allen, 2019) in our cohort of healthy human adults, despite their prevalence in mice. Whether these cells are present in other tissues and/or developmental stages is unclear. We also chose not to designate any subset as innate-like marginal zone B cells, as the marginal zone is an anatomical region that might contain multiple B cell subsets.

We identified six memory populations, confirming previous reports of phenotypic diversification after antigen recognition in mice and humans (Garraud et al., 2012). This should, however, not be confused with greater clonal diversity, as our immune repertoire analysis demonstrated that naive B cells had greater clonal diversity than their memory counterparts, as expected due to antigen selection and clonal expansion. We identified RB as a marker for memory cells and confirmed that RB^+^CD27^−^ cells have undergone SHM. The RB antibody clone MEM55 (used in this study) distinguishes B cell subsets because of differential glycosylation of RB, rather than differential usage of the RB isoform (Koethe et al., 2011). Unlike CD45RA and CD45RO splice isoforms in the switch of naive to memory T cells, the switch of naive to memory B cells identified here is accompanied by the post-translational modification of RB and therefore impossible to detect with mRNA sequencing, instead requiring a proteomic readout.

We also identified a CD19^hi^CD11c^+^ memory population that shares some common features with several populations that have been described in the context of autoimmunity, infection, and aging (Karnell et al., 2017). Within this population, we separated cells by CD27 expression and found an enrichment for T-bet and PD-1 within CD27^−^ CD19^hi^CD11c^+^ memory cells, similar to effector memory phenotypes seen in T cells (Lazarevic, Glimcher and Lord, 2013). Despite these altered expression profiles, we found no differences in metabolism, biosynthesis, or immune signaling between CD27^+^ and CD27^−^ fractions (data not shown). These cells lack CD184, have low expression of CD40, mixed expression of CD185, and were absent in both tonsil and lymph node, suggesting they might not participate in germinal center reactions, despite having the most potent response to activation in both the BCR and CD40 signaling pathways. This contrasts previous reports that have indicated cells with similar phenotypes are hyporesponsive to BCR and CD40 activation (Isnardi et al., 2010, Rakhmanov et al., 2009). This discrepancy might be explained by our use of high-dimensional clustering rather than binary gating, our shorter stimulation times, our quantification of signaling post-translational modifications as a readout, and our IgH-agnostic anti-kappa stimulation. Altogether, the increased frequency of this cell type in various disease states, its absence in secondary lymphoid organs, and its potent response to immune stimulation suggest that these cells are a population of effector memory cells.

CD95^+^ memory was also highly responsive to stimulation, suggesting they too might represent an effector memory population. CD95 can be expressed on activated lymphocytes as a mechanism for limiting inflammation because ligation of CD95 can lead to apoptosis (Daniel and Krammer, 1994) The ligation of CD95, however, does not exclusively result in apoptosis and it might be relevant in lymphocyte activation, survival, and proliferation (Peter et al., 2007), so rather than accelerating cell death, CD95 might be endowing these cells with increased potency and longevity. CD95 was, however, also expressed on GC B cells, so it is possible that CD95^+^ memory cells are simply recent germinal center emigrants that have retained expression of CD95.

Within the tonsil, we identified a population uniquely characterized by high expression of CD39. Although CD39 expression might be indicative of regulatory function, we would caution against that interpretation. The expression of CD73, a functionally related enzyme, was expressed by multiple populations that did not appear enriched for regulatory characteristics, so CD39 and CD73 might instead be part of larger expression programs that balance immune sensitivity and activation. Because these cells were exclusive to the tonsil, we did not label them as conventionally circulating memory, though phenotypically, they appear to be antigen experienced. Given that they shared phenotypic features with CD95^+^ memory, RB^−^ memory, and CD19^hi^CD11c^+^ memory, they might represent a precursor to these populations.

Further investigation of tissue-resident B cells is warranted because our study had a very limited cohort of lymphoid tissue donors and lymph node composition might vary by anatomical location. Future studies should utilize our phenotypic profiles to address compartmentalization and trafficking of B cell subsets with high-dimensional imaging. Our comprehensive characterization of surface phenotypes could also be paired with single-cell epigenetic, transcriptional, and/or immune repertoire profiles to give a more complete overview of cell identity. Furthermore, subsets should be prospectively isolated for further characterization of antibody secretion, plasma cell differentiation, TLR ligand sensitivity, cytokine production, and antigen presentation capabilities.

Here, our deep phenotypic profiling with multi-omic integration of numerous single-cell functional readouts in healthy individuals reveals the identity of new, more granular populations, comprehensively mapping B cell identity across blood and lymphoid tissues in the human. The quantitative assessment of the contribution of phenotype versus isotype usage across several cellular processes highlights the need for analysis beyond repertoire sequencing and isotype identity for understanding human B cell immune function. Our findings should serve as a resource for future studies investigating the humoral immune response in the context of vaccination or disease, as several populations and molecules described here may be crucial to understanding B-cell-mediated pathogenesis or protection.

## STAR★Methods

### Key Resources Table

**Table:** 

REAGENT or RESOURCE	SOURCE	IDENTIFIER
**Antibodies**		

Goat F(ab’)_2_ Anti-Human Kappa-UNLB	Southern Biotech	Cat#2062-01; RRID: AB_2795736
All antibodies used for cytometry	Various	Table S1

**Biological Samples**		

Leukocyte reduction chamber from human blood	Stanford Blood Center	stanfordbloodcenter.org
Whole blood from healthy donors	Obtained under informed consent with IRB approval	n/a
Human tonsil	Stanford Hospital, adult tonsilectomy from adults with obstructive sleep apnea	stanfordhealthcare.org
Human lymph node	Stanford Hosptial, biopsy sample of recovered lymphoma patient	stanfordhealthcare.org
Human bone marrow from healthy donors	AllCells	allcells.com

**Chemicals, Peptides, and Recombinant Proteins**		

Ficoll-Paque Plus	GE Healthcare	Cat#300-25
RPMI 1640 media	Thermo Fisher Scientific	Cat#21-870-092
GlutaMAX supplement	Thermo Fisher Scientific	Cat#35050-061
Fetal bovine serum USDA approved lot	Omega Scientific, Inc.	Cat#FB-01
Benzonase nuclease	Sigma-Aldrich	Cat#E1014-25KU
Recombinant human CD40L	BioLegend	Cat#591704
Human TruStain FcX (Fc Receptor Blocking Solution)	BioLegend	Cat#422302
Bovine Serum Album (BSA) Heat-shock Treated	Thermo Fisher Scientific	Cat#BP1600-100
Cell-ID Intercalator-Ir	Fluidigm	Cat#201192A
Cell-ID Cisplatin	Fluidigm	Cat#201064
Saponin from quillaja bark	Sigma-Aldrich	Cat#S7900-25G
Calibration Beads, EQ, Four Element	Fluidigm	Cat#201078
7-AAD Viability Staining Solution	BioLegend	Cat#420404
Streptavidin Particles Plus	BD Biosciences	Cat#557812

**Critical Commercial Assays**		

Cell-ID 20-Plex Pd Barcoding Kit	Fluidigm	Cat#201060
Maxpar X8 Antibody Labeling Kit	Fluidigm	Cat#201176B
IMag Cell Separation Magnet	BD Biosciences	Cat#552311
QIAamp DNA Micro Kit	QIAGEN	Cat#56304
immunoSeq Human B Cell - survey resolution	Adaptive	adaptivebiotech.com

**Deposited Data**		

Mass cytometry data	This study	flowrepository.org/id/FR-FCM-Z2MA flowrepository.org/id/FR-FCM-Z2MC

**Software and Algorithms**		

RStudio	Rstudio	rstudio.com
MATLAB	MathWorks	mathworks.com/products/MATLAB.html
Cytobank	Cytobank	cytobank.org
CellEngine	Primity Bio	cellengine.com

**Other**		

Custom code for analysis	This study	github.com/davidrglass/atlas

### Resource Availability

#### Lead contact

Further information and requests for resources and reagents should be directed to and will be fulfilled by the Lead Contact, Sean Bendall (bendall@stanford.edu).

#### Materials availability

This study did not generate new unique reagents

#### Data and code availability

The accession numbers for the mass cytometry data reported in this paper are Flow Repository: FR-FCM-Z2MA (surface screen) and FR-FCM-Z2MC (other). All associated code is available at github.com/davidrglass/atlas.

### Experimental Model and Subject Details

#### Human specimens

Deidentified human blood (n = 20) and bone marrow (n = 3) were obtained from healthy adult donors (Stanford Blood Center, AllCells). Deidentified, discarded surgical tonsil samples from healthy adult patients undergoing tonsillectomy for obstructive sleep apnea were collected (n = 3). The lymph node (n = 1) was excised from a 60 year old woman with increased FDG uptake on PET scan and a history of diffuse large B cell lymphoma, last treated with R-CHOP four years prior. It showed normal follicular architecture and immunophenotype by histology and flow cytometry, respectively. The patient received no subsequent therapy and no radiographic evidence of lymphoma or progression was seen in the two years since. All samples were obtained under informed consent and in accordance with Stanford’s Institutional Review Board.

### Method Details

#### Peripheral blood and bone marrow processing

Mononuclear cells (MCs) were isolated from Trima Accel leukocyte reduction system (LRS) chambers or heparinized tubes using Ficoll-Paque Plus (GE Healthcare) density gradient centrifugation according to the manufacturer’s instructions. For long-term storage (surface screen and tissue phenotyping only), MCs were resuspended in fetal bovine serum (FBS; Omega Scientific, Inc.) with 10% DMSO, slowly cooled to −80°C, and stored in liquid nitrogen at a density of 1-5 × 10^7^ cells/mL. Cryopreserved MCs were thawed into cell culture medium (CCM; RPMI 1640 containing 10% FBS, and GlutaMAX; Thermo Fisher Scientific) supplemented with 25cU/mL benzonase (Sigma-Aldrich). and pelleted for 5cmin at 250c*g*. Where indicated, cells underwent magnetic lineage depletion according to the manufacturer’s instructions using BD Streptavidin Particles Plus and the BD IMag Cell Separation Magnet (BD Biosciences) with biotinylated anti-CD3 (surface screen samples) or a cocktail of biotinylated antibodies consisting of CD3, CD7, CD15, CD33, CD56, CD61, and CD235ab (other samples). The biotinylated antibody cocktail was detected by labeled anti-biotin (mass cytometry) and streptavidin (FACS) and further depleted *in silico*.

#### Tissue processing

After lymph node excision, a fresh portion was placed in RPMI and refrigerated until it could be minced into ∼1-3 mm fragments with a clean blade. Fragments were resuspended in 3 mL DPBS and refrigerated. After diagnostic testing was complete, a portion of the remainder was filtered through 35 μm nylon mesh, centrifuged at 250 x g for 5 min, resuspended in 10% FBS at approximately ∼5 × 10^6^ cells/aliquot, slowly cooled to −80°C, and then stored in liquid nitrogen. Whole tonsil pairs were collected in saline and processed immediately. Tonsils were disinfected in an antibiotic cocktail for 30 min, rinsed with saline, and any cauterized tissue was removed. The remaining healthy tissue was cut into small pieces (approximately 2mm x 2mm x 5mm) and pressed through a 100um nylon mesh strainer using a syringe plunger. The strainer was rinsed with serum-containing media (RPMI1640 with 10% FBS) and the released cells were washed two times. After enumeration, cells were cryopreserved in approximately 50-100 × 10^6^ cells/aliquot in FBS + 10% DMSO at −80°C and transferred to long-term storage in liquid nitrogen the next day.

#### Metabolism, biosynthesis activity, and stimulation assays

PBMCs were rested in 37°C 5% CO_2_ incubator for 30 min (biosynthesis activity assays), 1 h (metabolism assays), or 2 h (stimulation assays) at 5 × 10^6^ cells/mL in CCM. Metabolism samples were fixed in 1.6% PFA in PBS for 10 min and then palladium barcoded as previously described (Zunder et al., 2015). After rest, biosynthesis activity assay samples were spiked with 2 mM BRU and 10 μg/mL puromycin and incubated for an additional 30 min (Kimmey et al. 2019) and then fixed and palladium barcoded. Stimulation samples were resuspended in CCM with 3.3 mM H_2_O_2_ (Irish et al., 2010) and specified doses of anti-kappa F(ab’)_2_ (Southern Biotech) and CD40L (BioLegend) for 10 min and then fixed and palladium barcoded.

#### Mass cytometry antibody conjugation, staining, and data acquisition

Antibody conjugation, staining, and data acquisition were performed as previously described (Hartmann, Simonds and Bendall, 2018). Briefly, metal-isotope labeled antibodies used in this study were conjugated using the MaxPar X8 Antibody Labeling kit per manufacturer instruction (Fluidigm), or were purchased from Fluidigm pre-conjugated. Each conjugated antibody was quality checked and titrated to optimal staining concentration using a combination of primary human cells and/or cancer cell lines (Figure S1C). Tissue samples were live-cell barcoded as previously described (Hartmann, Simonds and Bendall, 2018). Cells were suspended in TruStain FC blocker for 10 min at RT and washed in cell staining media (CSM: PBS with 0.5% BSA and 0.02% sodium azide and benzonase 25x10^8^ U/mL (Sigma)) prior to staining. All surface staining was performed in CSM for 30 min at RT. Cells were washed in CSM and resuspended in cisplatin for 5 min to label non-viable cells (Sigma, 0.5 μM final concentration in PBS). Cells were washed in CSM and fixed with 1.6% PFA in PBS for 10 min at RT and (if intracellularly stained) washed in CSM and permeabilized with MeOH for 10 min on ice. Intracellular and anti-biotin staining was performed in CSM for one h at RT. Before acquisition, samples were washed in CSM and resuspended in intercalation solution (1.6% PFA in PBS, 0.02% saponin (Sigma) and 0.5 μM iridium-intercalator Fluidigm)) for 1 h at RT or overnight at 4°C. Before acquisition, samples were washed once in CSM and twice in ddH_2_O. All samples were filtered through a 35 μm nylon mesh cell strainer, resuspended at 1 × 10^6^ cells/mL in ddH_2_O supplemented with 1x EQ four element calibration beads (Fluidigm), and acquired on a CyTOF2 mass cytometer (Fluidigm). Barcoded samples were acquired using the Super Sampler injection system (Victorian Airship).

#### FACS sorting, gDNA extraction, and immune repertoire sequencing

PBMCs were lineage depleted and Fc blocked as described above. Cells were surface stained (Table S1) in CSM in the dark for 30 min on ice and then washed in CSM. Prior to data acquisition, cell suspensions were spiked with 7-AAD (BioLegend) to label non-viable cells. Non-transitional/non-plasma B cells were gated as singlet, viable, CD45^+^, lin^-^, CD19^+^, CD38^lo/-^ and then sorted from the four quadrants of the CD27 x RB biaxial plot using a BD FACS Aria II (BD Biosciences). Approximately 100,000 cells per subset per donor were sorted into tubes. Cells were lysed and gDNA was extracted using the QIAamp DNA Micro kit (QIAGEN) according to the manufacturer’s instructions. DNA was frozen and shipped to Adaptive Biotechnologies for IgH library preparation and next-generation sequencing using the immunoSEQ Assay (Adaptive).

### Quantification and Statistical Analysis

#### Mass cytometry data pre-processing

Acquired samples were bead-normalized using MATLAB-based software as previously described (Finck et al., 2013). Where applicable, barcoded data was debarcoded using MATLAB-based software (Zunder et al., 2015). Normalized data was then uploaded onto the Cytobank analysis platform for gating (Figures 1B and S1A; Kotecha, Krutzik and Irish, 2010). Gated data was downloaded and further processed with the R programming language (http://www.r-project.org) and Bioconductor (http://www.bioconductor.org) software. Data was transformed with an inverse hyperbolic sine (asinh) transformation with a cofactor of 5. Molecules on the screen compromised by bleed from other channels or by any other technical considerations were removed. Conserved molecules from the surface screen were quantile normalized by donor to correct for technical variation between mass cytometry runs. Peak normalization (alignment of mode of positive population) of each molecule was applied to normalize samples from different donors in Figure 4 only, as those samples were not barcoded and stained in the same tube (Figure S2A). As the tissue samples (Figure 6) were processed and collected in two batches; batches were normalized to produce equal 99.9^th^ percentile expression of each molecule within the peripheral blood samples. For all experiments, each molecule was scaled to the 99.9^th^ percentile of expression of all cells in that experiment for comparability between parameters. Individual cell quantifications from all donors were pooled together for all analyses except where noted to utilize all observations acquired.

#### Surface screen thresholding

To avoid subjectivity in determining whether each molecule was present or absent on B cells, we set a uniform threshold for positivity, mandating that the 99.9^th^ percentile of expression in B cells for each molecule was at least 40 raw counts (2.78 asinh-transformed value) to be considered positive. The cutoff was set at a high value to prevent inclusion of false positives with high background staining, at the expense of enriching for false negatives. This approach provided confidence that all molecules assessed as positive were truly present on B cells, while those assessed as negative were either absent on B cells or were present at low levels. The 99.9^th^ percentile was used instead of median values for thresholding, in order to capture molecules expressed by a small subset of B cells (e.g., IgA) and not just those uniformly expressed by all B cells (e.g., beta-2 microglobulin, Figure 1C). Furthermore, with a mean of 245 thousand B cells assessed with each mass cytometry panel in the screen, a molecule was on average only considered positive if ∼245 cells express the target at a high level. The dataset could be reanalyzed to identify more rarely- or more lowly-expressed markers.

#### Ig quantification

To quantify surface Ig (surface stain, Figures 4 and 6) and total Ig (intracellular stain, Figure 5) across IgH isotypes, Igκ and Igλ light chains were independently measured on two different mass cytometry channels. The two distributions were peak normalized within each experiment (Figure S2C) and then each cell was assigned a new parameter, surface or total Ig, calculated as the pairwise maximum of the two peak normalized light chain channels. As B cells can only express a single light chain isotype, the pairwise maximum represents true signal, while the pairwise minimum represents noise and can therefore be discarded. This approach provides an independent quantification of Ig expression for single cells that is not biased by antibodies with differing affinities to the various IgH isotypes.

#### Dimensionality Reduction

To visualize co-expression of molecules measured on different cells in the surface screen, cells stained with different mass cytometry panels were plotted together on a single UMAP plot using the umap package in R (Figures 3A and 3B). 2,500 cells from each of the 12 mass cytometry panels were randomly subsampled and used to generate a UMAP plot (uwot package) based on the expression of molecules positive on B cells that are conserved in all panels: CD45, CD19, CD24, CD38, CD27, IgM, and IgD. This ensured that cells of similar phenotype localized into similar coordinates, facilitating qualitative assessment of molecule co-expression between molecules that were not measured on the same cell. Color overlay of molecule expression for a given molecule used only cells from the screen on which that molecule was measured for the visualization. For visualization of surface molecule expression of our meta-clusters, 1,000 cells were randomly subsampled from each of the ten B cell subsets and used to generate a UMAP plot (Figure 4D). The plot was generated based on expression of all phenotypic molecules. Isotype was not used to generate the map to prevent artificial separation of phenotypically similar cells. Subsampling by B cell subset facilitated visualization of heterogeneity within and between populations without the map being dominated by the most abundant populations. The same approach was taken for the UMAP in Figure 6, with the addition of an equal subsampling by tissue before an subsampling by subset (840 cells per subset).

#### Clustering

For initial subset discovery (Figure 4) cells were over-clustered into 169 clusters using FlowSOM with all molecules as input. Clusters were then hierarchically clustered as either “antigen-inexperienced” or “antigen-experienced” based on median expression of RB, CD27, CD305, CD44, and CD11c. Antigen-inexperienced clusters were then hierarchically clustered into three subsets: (1) Transitional, (2) CD73^-^ Naive, and (3) CD73^+^ Naive, based on expression of CD38, CD79b, and CD73. Antigen-experienced clusters were hierarchically clustered into four subsets: (1) Plasma, (2) CD95^+^ Memory, (3) CD19^hi^ CD11c^+^ Memory, and (4) other memory based on expression of CD20, CD268, CD95, and CD11c. Other memory clusters were hierarchically clustered into two subsets: (1) RB^-^ Memory and (2) RB^+^ Memory based on expression of RB. RB^+^ Memory was then hierarchically clustered into three subsets: (1) CD27^-^ Memory, (2) RB^+^ CD27^+^ CD73^-^ Memory, and (3) RB^+^ CD27^+^ CD73^+^ Memory based on expression of CD27 and CD73. This entire procedure resulted in the identification of ten unique subsets. This approach was selected for several reasons: A) The initial over-clustering step groups phenotypically similar cells based on high-dimensional data and prevents arbitrarily drawing lines between overlapping populations based on a single molecule, as in canonical gating schemes. B) Hierarchical clustering allows segregation of cells into large groups (antigen-inexperienced versus antigen-experienced) before further subsetting. This mirrors canonical gating, where T cells are segregated from B cells before expression of CD4 or CD8 is considered. C) This approach allows us to use specific molecules to subset specific groups of cells. Once we have determined that expression of a parameter is uniform in a group (e.g., RB in naive cells), the use of that molecule as a clustering parameter will only add noise to the model. Instead, we selected molecules for each group where there was meaningful differential expression (e.g., CD73 in naive cells).

For subsequent subsetting of other datasets (Figures 5 and 6), cells were over-clustered with surface and intracellular Ig molecules and then manually segregated into subsets in a gating scheme similar to Figures S2B and S6H. This ensured consistency of classification between datasets. For signaling data, only unstimulated cells were clustered and classified so that Ig levels could be used in the initial clustering step. After crosslinking, Ig levels diminish, so clustering based on these molecules causes a segregation of cells based on stimulation dose. Stimulated cells were instead classified into subsets based on a KNN (k = 3) classifier trained on unstimulated data, using only Euclidean distance of surface molecule expression, which is not altered by the short stimulation.

#### GO quantifications

“Biological Processes” gene ontology annotations for all molecules on the surface screen were compiled from UniProt (https://www.uniprot.org). This resulted in over 2,000 unique annotations, so GO terms were collapsed into 30 parent terms using the Generic GO Term Mapper (Figure S1B) (https://go.princeton.edu/cgi-bin/GOTermMapper).

#### Immune repertoire sequencing pre-processing

Templates per sequence (number of unique cells with identical IgH sequences) was determined by Adaptive Biotechnology based on number of sequencing reads normalized to spiked-in controls of “artificial” IgH sequences. Sequences with less than ten reads were eliminated from the analysis. The Immcantation pipeline (Vander Heiden et al., 2014; Gupta et al., 2015) was used for downstream processing and analysis. V and J gene usage was determined using IgBlast (Ye et al., 2013) on the IMGT database (Lefranc et al., 2015) and corrected by Bayesian inference of each donor’s genotype. Clonal lineages assignments of sequences were made with the following requirements: same donor, identical V and J gene usage, identical CDR3 length, and a hamming distance to another member of the lineage beneath the set threshold. This distance threshold was determined for each donor by fitting a generalized mixture model based on the density plot of the hamming distance of the CDR3 to its nearest neighbor for each sequence. This creates a bimodal distribution of sequences with a clonal relative (lower peak) and sequences without a clonal relative (higher peak). The intersection of the two fitted gaussians was set as the distance threshold for clonal membership. Germline sequences were inferred for each clonal lineage and silent and non-silent mutations outside of the CDR3 were quantified as deviations from the inferred germline.

#### Immune repertoire analyses

Mutation frequency was calculated as the frequency of mutations from the reconstructed germline sequence to input sequence (Figure 3G). The D region and N/P nucleotides were excluded from the calculation as they are difficult to accurately call and reconstruct. Mutations were binned as either silent (no amino acid change) or replacement (amino acid change). Sequence diversity was calculated using the general form of the diversity index (Hill, 1973) over a range of orders (Figure 3H). 95% confidence intervals were generated by 200 bootstrap resampling calculations, as previously described (Gupta et al., 2015). For clonal analysis, all sequences were labeled by their population of origin and then these labels were then randomly permuted (Figure 3I). For each population label, the frequency in which a sequence from population X shared a clonal lineage with a sequence from population Y was quantified and then repeated for all combinations of the four populations. This calculation is asymmetric as the frequency in which a sequence from population X shares a lineage with a sequence from population Y is not the same as the frequency in which a sequence from population Y shares a lineage with a sequence from population X. This process was repeated 200 times to create a null distribution and then z-scores of the frequencies were derived for the observed data using the original population labels (Figure 3J). This approach shares some features of significance analysis of microarrays (SAM) analysis (Tusher, Tibshirani and Chu, 2001), where null distributions are also created through permutation analysis and scores are assigned on the basis of changes in expression relative to the standard deviation.

#### Statistics

All statistical tests for differences in distribution of molecules between B cell subsets from mass cytometry data were performed on equally subsampled populations using the KS test. This non-parametric test determines the equality of two continuous distributions and is sensitive to both changes in mean and shape of a distribution. It can therefore detect if even a fraction of a subset has a change in expression compared to the reference population. All P values were corrected by the Bonferroni method, the most conservative of multiple hypothesis correction approaches, and only considered significant if the adjusted p < 0.005, rather than the standard p < 0.05. These rigorous statistical conditions were chosen to prevent inclusion of false positives at the expense of enriching for false negatives. This approach provides confidence that our differential marker expression analysis is reflective of significant biological differences.

For comparisons of mutation frequencies between sorted B cell populations (Figure 3G) and analysis of B cells positive for T-bet (Figure S4B), the Wilcoxon rank sum test was performed with Bonferroni correction, and only considered significant if p < 0.005. To determine the contribution of phenotype and isotype to various processes (Figures 4I and 5J), a multiple linear regression model was used for each response. Each observation (cell) was labeled with two discrete predictors: phenotype (B cell subset membership determined by clustering) and isotype (determined by manual gating). These two predictors were used to regress the continuous expression value of the desired response variable (e.g., CD79b expression). The relative contribution to variance explained by each predictor was calculated using the “lmg” metric of the relaimpo R package (Grömping, 2015).

## Supplemental Information

Document S1. Figures S1-S8

Table S1: Cytometry panels used in the study, related to Figures 1, 3, and 4–6

Document S2. Article plus Supplemental Information
