# Supplementary material for: An Integrated Multi-omic Single-Cell Atlas of Human B Cell Identity
Source: Immunity. 2020 Jul 14;53(1):217–232.e5. doi: 10.1016/j.immuni.2020.06.013 (PMC7369630; doi:10.1016/j.immuni.2020.06.013)
Supplement: Document S1. Figures S1-S8 [file mmc1.pdf]

Immunity, Volume *53*

Supplemental Information

An Integrated Multi-omic Single-Cell

Atlas of Human B Cell Identity

David R. Glass, Albert G. Tsai, John Paul Oliveria, Felix J. Hartmann, Samuel C. Kimmey, Ariel A. Calderon, Luciene Borges, Marla C. Glass, Lisa E. Wagar, Mark M. Davis, and Sean C. Bendall

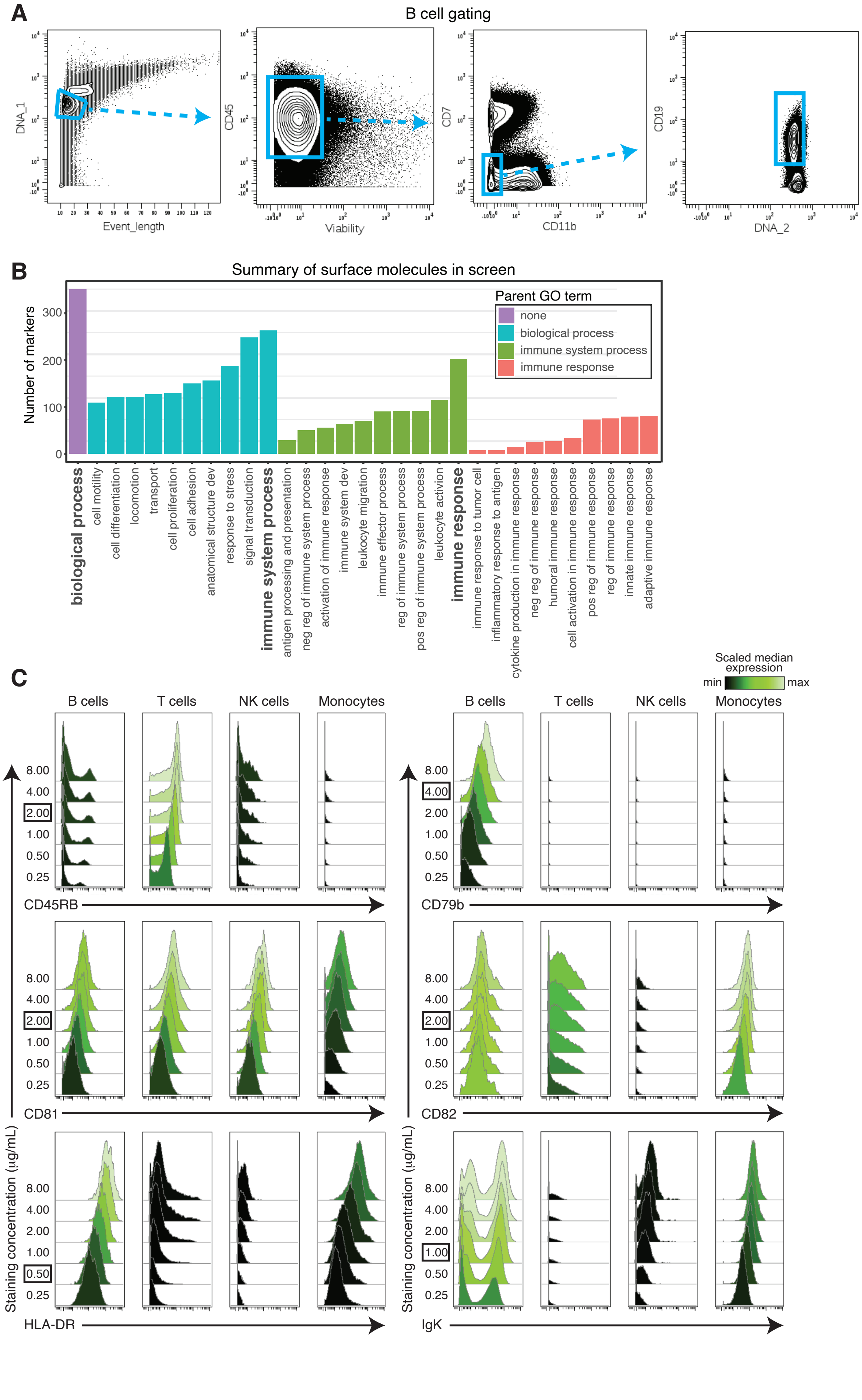
A
B cell gating
DNA_1
Event_length
CD45
Viability
CD7
CD11b
CD19
DNA_2
B
Summary of surface molecules in screen
Number of markers
Parent GO term
none
biological process
immune system process
immune response
biological process
cell motility
cell differentiation
locomotion
transport
cell proliferation
cell adhesion
anatomical structure dev
response to stress
signal transduction
immune system process
antigen processing and presentation
neg reg of immune system process
activation of immune response
immune system dev
leukocyte migration
immune effector process
reg of immune system process
pos reg of immune system process
leukocyte activion
immune response
immune response to tumor cell
inflammatory response to antigen
cytokine production in immune response
neg reg of immune response
humoral immune response
cell activation in immune response
pos reg of immune response
reg of immune response
innate immune response
adaptive immune response
C
Scaled median expression
min
max
B cells
T cells
NK cells
Monocytes
CD45RB
CD79b
CD81
CD82
HLA-DR
IgK
Staining concentration (μg/mL)
8.00
4.00
2.00
1.00
0.50
0.25

Figure S1: Surface screen gating, summary, and quality control - related to Figure 1

A) Representative plots from one donor of the gating strategy for total B cells in the surface screen. **B)** Quantification of the number of surface molecules in the surface screen that were associated with specific GO annotations. **C)** Representative plots of surface screen antibody staining concentration titrations of healthy PBMCs, arranged by immune population (columns) and staining concentration (rows, μg/mL). Staining concentration used in the screen is boxed. All populations are defined as CD45+ lin-. Additionally, B cells are CD19+, T cells are CD3+, NK cells are CD56+, and monocytes are CD14+.

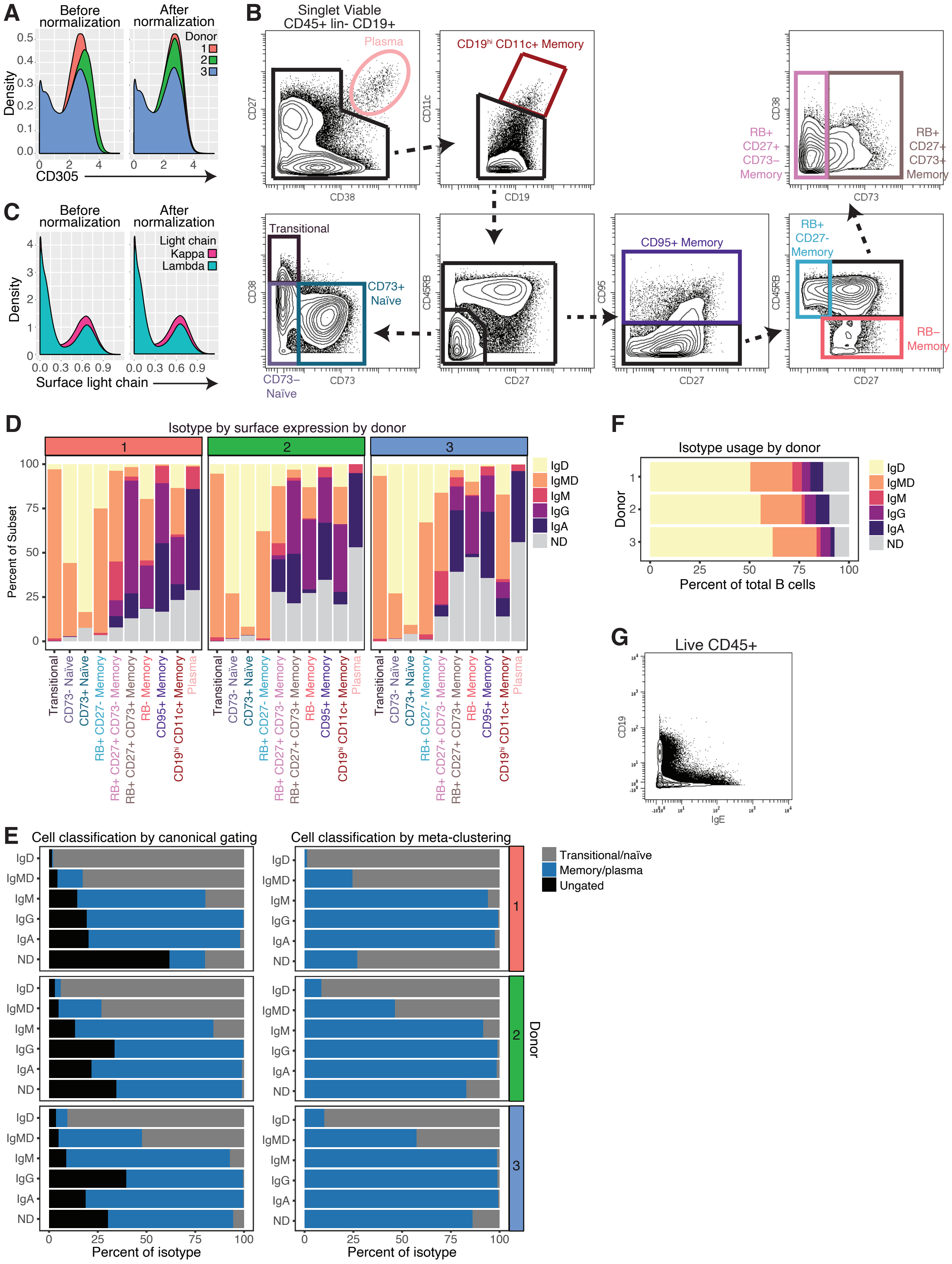
A
Before normalization
After normalization
Donor
1
2
3
Density
CD305
B
Singlet Viable CD45+ lin- CD19+
Plasma
CD19hi CD11c+ Memory
CD27
CD38
CD11c
CD19
RB+ CD27+ CD73– Memory
RB+ CD27+ CD73+ Memory
CD73
Transitional
CD73+ Naïve
CD73– Naïve
CD45RB
CD95
CD95+ Memory
RB+ CD27- Memory
RB– Memory
C
Light chain
Kappa
Lambda
Surface light chain
D
Isotype by surface expression by donor
Percent of Subset
IgD
IgMD
IgM
IgG
IgA
ND
Transitional
CD73- Naïve
CD73+ Naïve
RB+ CD27- Memory
RB+ CD27+ CD73- Memory
RB+ CD27+ CD73+ Memory
RB- Memory
CD95+ Memory
CD19hi CD11c+ Memory
Plasma
E
Cell classification by canonical gating
Cell classification by meta-clustering
Transitional/naïve
Memory/plasma
Ungated
Percent of isotype
Donor
F
Isotype usage by donor
Percent of total B cells
G
Live CD45+
CD19
IgE

Figure S2: Quality control, data processing, and individual donor contributions to phenotypic characterization - related to Figure 4.

A) Representative density plots of CD305 expression for total B cells of three donors (colors) before and after peak normalization. **B)** Representative plots from a single donor of the gating strategy for B cell subsets. **C)** Representative density plots of light chain isotype expression (colors) for total B cells pooled from three donors before and after peak normalization. **D)** IgH isotype usage by subset and donor. ND denotes “not determined”; IgMD denotes co-expression of IgM and IgD. **E)** Subset composition by isotype for each donor as determined by canonical gating or meta-clustering **F)** IgH isotype usage by donor. ND denotes “not determined”; IgMD denotes co-expression of IgM and IgD. **G)** Representative contour plot from one donor from the surface screen of total CD45+ cells showing low co-occurrence of IgE and CD19.

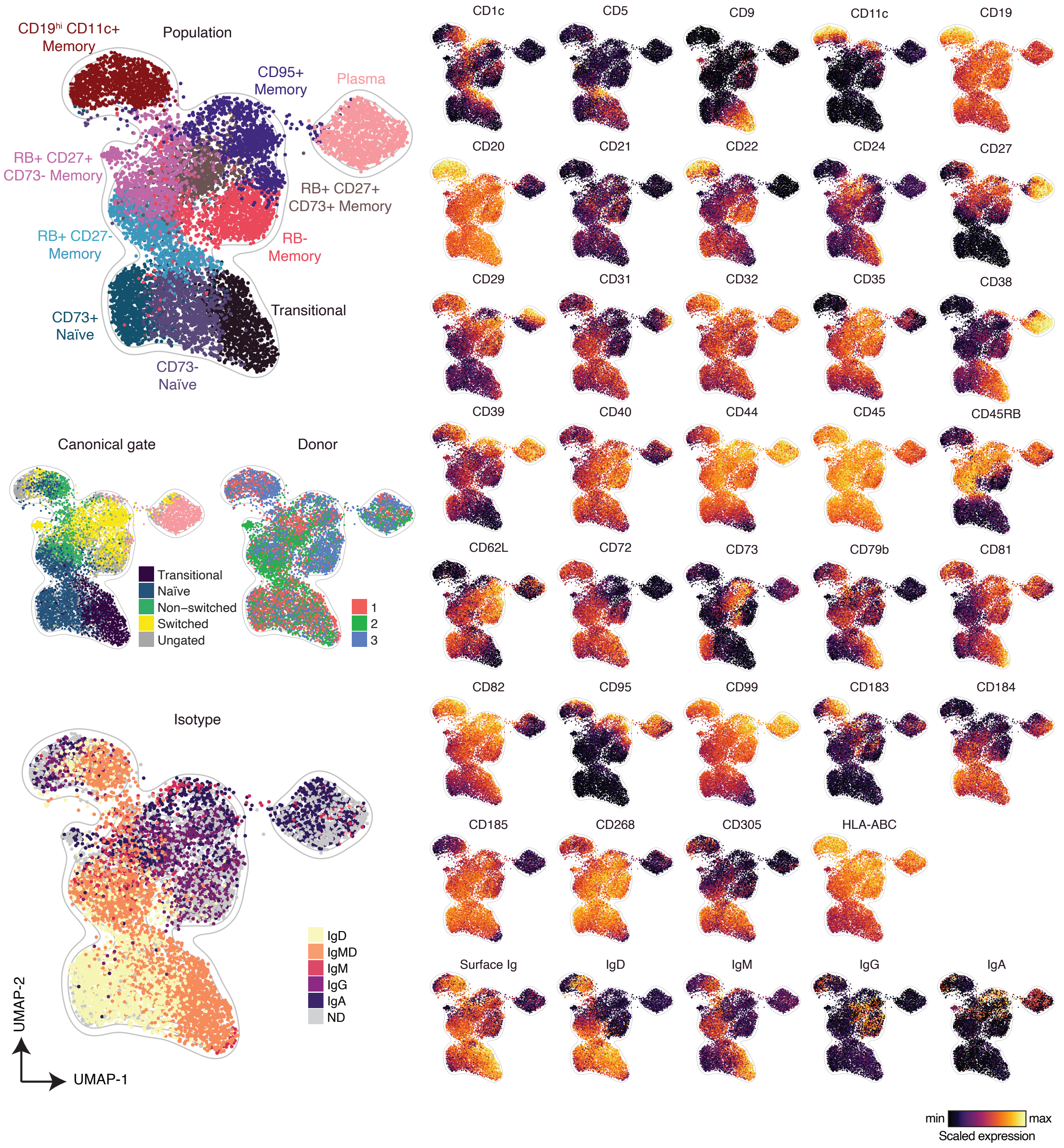

CD19hi CD11c+ Memory
Population
CD95+ Memory
Plasma
RB+ CD27+ CD73- Memory
RB+ CD27+ CD73+ Memory
RB+ CD27- Memory
RB- Memory
CD73+ Naïve
Transitional
CD73- Naïve
Canonical gate
Donor
Transitional
Naïve
Non−switched
Switched
Ungated
1
2
3
Isotype
IgD
IgMD
IgM
IgG
IgA
ND
UMAP-2
UMAP-1
CD1c
CD5
CD9
CD11c
CD19
CD20
CD21
CD22
CD24
CD27
CD29
CD31
CD32
CD35
CD38
CD39
CD40
CD44
CD45
CD45RB
CD62L
CD72
CD73
CD79b
CD81
CD82
CD95
CD99
CD183
CD184
CD185
CD268
CD305
HLA-ABC
Surface Ig
IgD
IgM
IgG
IgA
min
max
Scaled expression

Figure S3: Comprehensive phenotypic profiling of B cell subsets by UMAP - related to Figure 4.

UMAP plot generated from an equal subsampling of 1000 cells from each subset using only phenotypic (not isotypic) molecules. UMAP coordinates are identical to Figure 4D.

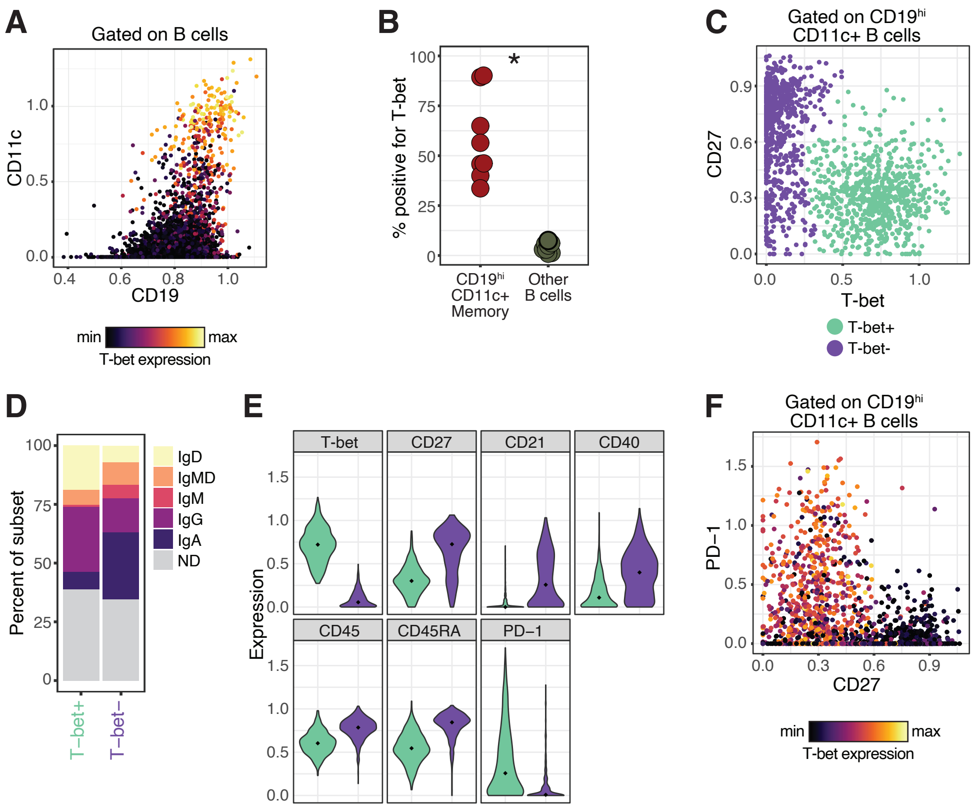
A
Gated on B cells
CD11c
CD19
min
max
T-bet expression
B
% positive for T-bet
*
CD19hi CD11c+ Memory
Other B cells
C
Gated on CD19hi CD11c+ B cells
CD27
T-bet
T-bet+
T-bet-
D
Percent of subset
IgD
IgMD
IgM
IgG
IgA
ND
T−bet+
T−bet−
E
Expression
T-bet
CD27
CD21
CD40
CD45
CD45RA
PD−1
F
Gated on CD19hi CD11c+ B cells
PD−1
CD27
min
max
T-bet expression

Figure S4: Further phenotypic characterization of CD19^{hi} CD11c+ Memory from a public dataset – related to Figure 4:

A) Biaxial of B cells from a representative healthy donor **B)** Percent of CD19^{hi} CD11c+ Memory cells and other B cells positive for T-bet in healthy donors. Star indicates significance ($p<0.005$, Wilcoxon rank sum test, n=8). **C)** Biaxial of donor-pooled (n=3) CD19^{hi} CD11c+ Memory cells, colored by T-bet positivity, as determined by hierarchical clustering of the two axes. **D)** IgH isotype usage by subset. ND denotes “not determined”; IgMD denotes co-expression of IgM and IgD. **E)** Violin plots of significantly differentially-expressed molecules (KS test, $p<0.005$, Bonferroni correction). Diamond denotes median. **F)** Biaxial of donor-pooled (n=3) CD19^{hi} CD11c+ Memory cells.

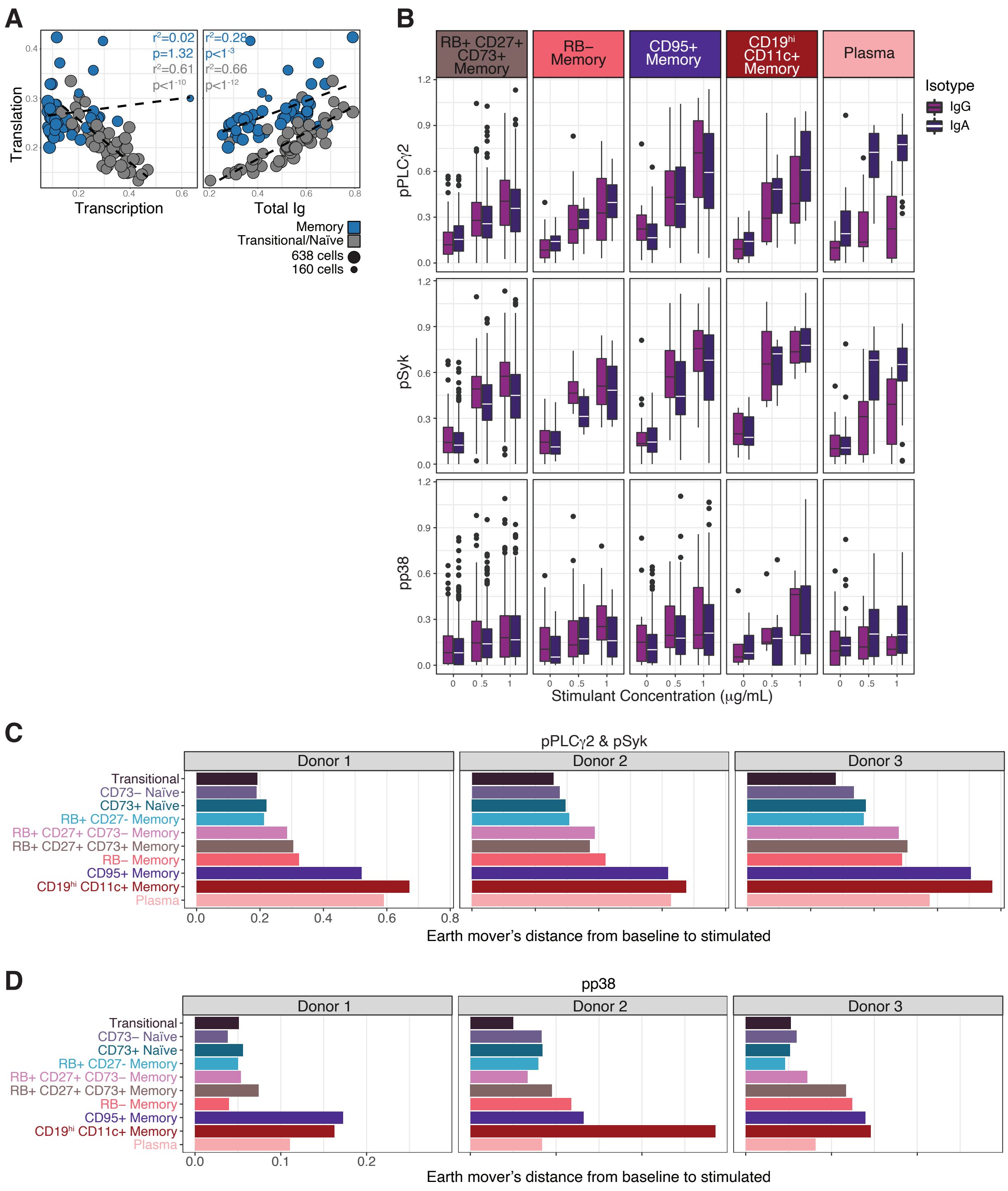

A
Translation
Transcription
Total Ig
r²=0.02
p=1.32
r²=0.61
p<1⁻¹⁰
r²=0.28
p<1⁻³
r²=0.66
p<1⁻¹²
Memory
Transitional/Naïve
638 cells
160 cells
B
RB+ CD27+ CD73+ Memory
RB– Memory
CD95+ Memory
CD19hi CD11c+ Memory
Plasma
Isotype
IgG
IgA
pPLCγ2
pSyk
pp38
Stimulant Concentration (μg/mL)
C
pPLCγ2 & pSyk
Donor 1
Donor 2
Donor 3
Transitional
CD73– Naïve
CD73+ Naïve
RB+ CD27- Memory
RB+ CD27+ CD73– Memory
RB+ CD27+ CD73+ Memory
RB– Memory
CD95+ Memory
CD19hi CD11c+ Memory
Plasma
Earth mover's distance from baseline to stimulated
D
pp38
Donor 1
Donor 2
Donor 3
Earth mover's distance from baseline to stimulated

Figure S5: Biosynthesis correlations, mature isotype signaling responses, and individual donor signaling responses – related to Figure 5

A) Biaxial plots of median expression of clusters (generated using FlowSOM – also used for initial over-clustering before subset assignment), colored as either memory (blue) or transitional/naïve (grey). Circle size indicates number of cells in cluster. Statistics and lines were calculated from simple linear regression models. **B)** Boxplots of the expression of three signaling molecules at three different doses (x-axis) segregated by isotype (colors) and by phenotype (columns). Only phenotypes that were > 3% IgG+ and > 3% IgA+ were included in visualization **C)** Quantification of earth mover's distance from baseline samples to stimulated samples (1 µg/mL) for pPLCγ2 and pSyk for individual donors. **D)** Quantification of earth mover's distance from baseline samples to stimulated samples (1 µg/mL) for pp38 for individual donors.

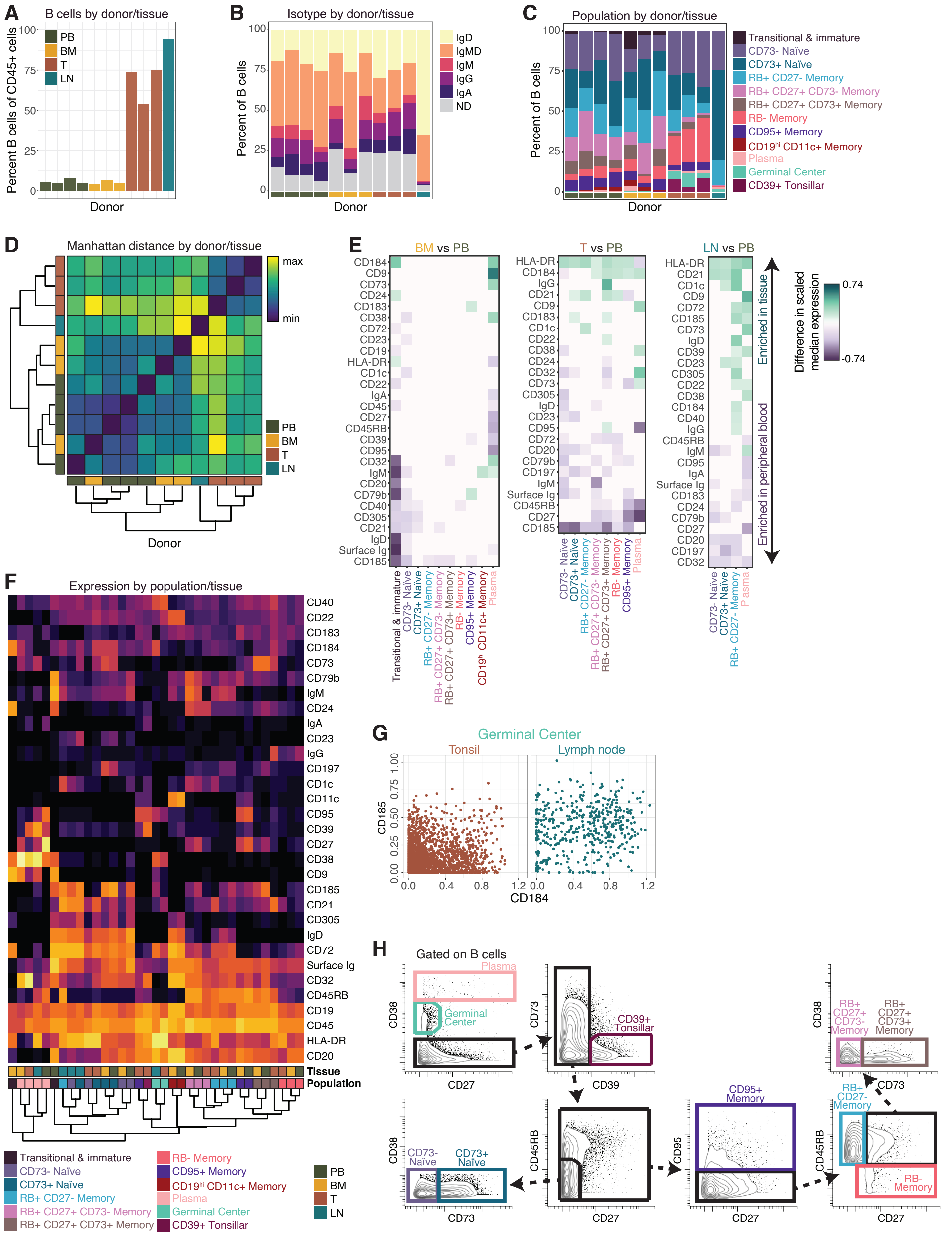

A
B cells by donor/tissue
Percent B cells of CD45+ cells
Donor
PB
BM
T
LN
B
Isotype by donor/tissue
Percent of B cells
Donor
IgD
IgMD
IgM
IgG
IgA
ND
C
Population by donor/tissue
Percent of B cells
Donor
Transitional & immature
CD73- Naïve
CD73+ Naïve
RB+ CD27- Memory
RB+ CD27+ CD73- Memory
RB+ CD27+ CD73+ Memory
RB- Memory
CD95+ Memory
CD19hi CD11c+ Memory
Plasma
Germinal Center
CD39+ Tonsillar
D
Manhattan distance by donor/tissue
max
min
Donor
E
BM vs PB
T vs PB
LN vs PB
Enriched in tissue
Enriched in peripheral blood
Difference in scaled median expression
0.74
-0.74
F
Expression by population/tissue
Tissue
Population
G
Germinal Center
Tonsil
Lymph node
CD185
CD184
H
Gated on B cells
Plasma
Germinal Center
CD39+ Tonsillar
CD73- Naïve
CD73+ Naïve
CD95+ Memory
RB+ CD27- Memory
RB- Memory
RB+ CD27+ CD73- Memory
RB+ CD27+ CD73+ Memory
CD38
CD27
CD73
CD39
CD45RB
CD95

Figure S6: individual donor contributions to phenotypic characterization, differential expression analysis, tissue-specific subset expression profiles and tissue gating scheme – related to Figure 6:

A) Percent B cells of CD45+ cells for each donor, colored by tissue. **B)** IgH isotype usage by donor and tissue. ND denotes "not determined"; IgMD denotes co-expression of IgM and IgD. **C)** Subset composition by donor and tissue. **D)** Pairwise Manhattan distance between each donor, ordered by hierarchical clustering. The distance was calculated based on the proportion of B cells in each subset for each donor. **E)** Difference in median expression between peripheral blood and indicated tissue for each subset and molecule. Only comparisons with at least 150 cells in each tissue/subset and an absolute difference > 0.1 and a p-value < 0.005 by KS test after Bonferroni correction are plotted – all other comparisons are absent from the heatmap or colored white. Rows are ordered by row-mean, resulting in an organization of the heatmap in which molecules enriched in peripheral blood appear at the top and those enriched in tissue appear at the bottom. **F)** Median expression for each molecule segregated by subset and tissue. Only subsets with at least 150 cells in a given tissue are plotted. Both axes are organized by hierarchical clustering. **G)** Representative plots from a single donor of the gating strategy for B cell subsets within tonsil.

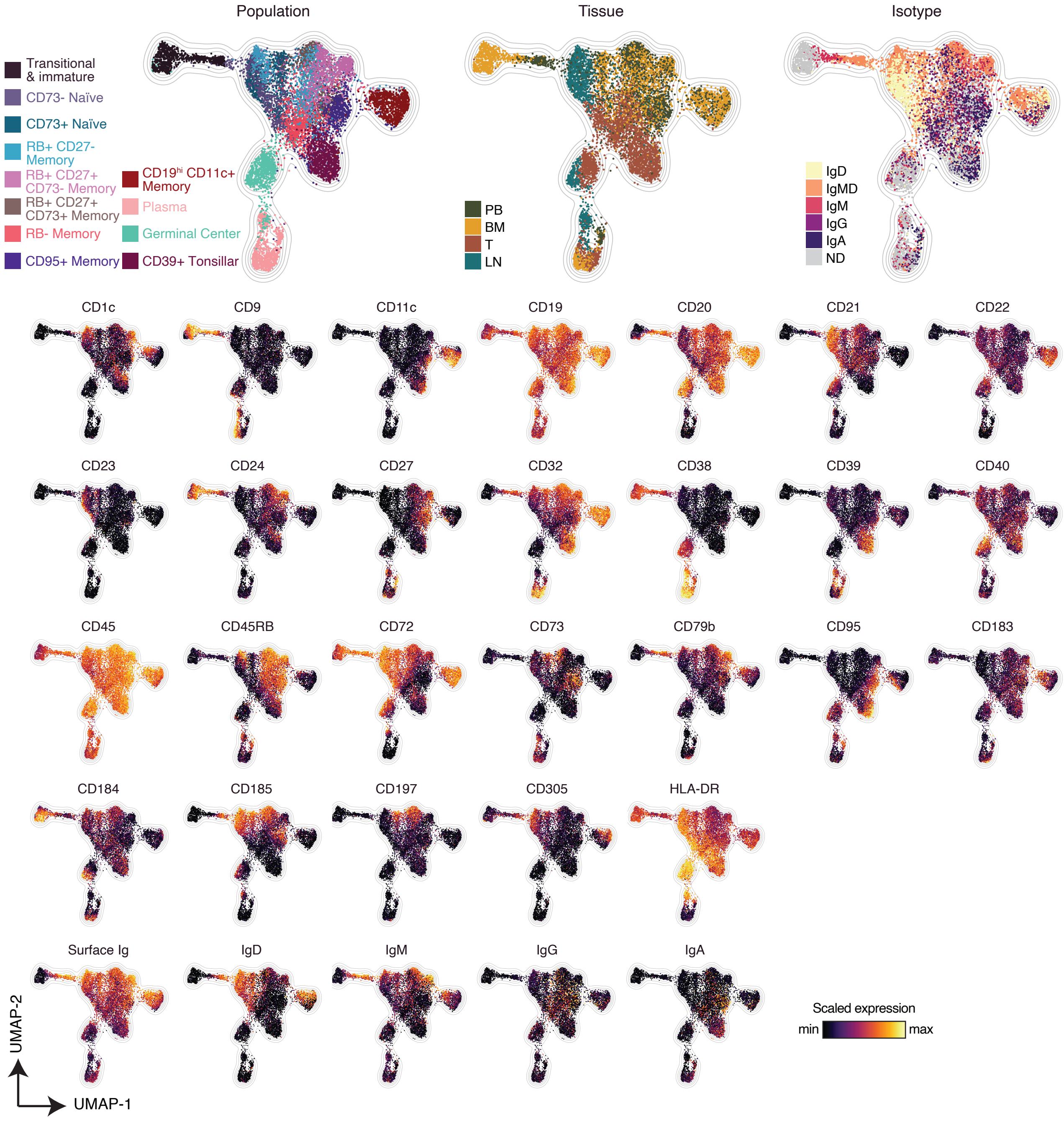
Population
Tissue
Isotype
Transitional & immature
CD73- Naïve
CD73+ Naïve
RB+ CD27- Memory
RB+ CD27+ CD73- Memory
RB+ CD27+ CD73+ Memory
RB- Memory
CD95+ Memory
CD19hi CD11c+ Memory
Plasma
Germinal Center
CD39+ Tonsillar
PB
BM
T
LN
IgD
IgMD
IgM
IgG
IgA
ND
CD1c
CD9
CD11c
CD19
CD20
CD21
CD22
CD23
CD24
CD27
CD32
CD38
CD39
CD40
CD45
CD45RB
CD72
CD73
CD79b
CD95
CD183
CD184
CD185
CD197
CD305
HLA-DR
Surface Ig
IgD
IgM
IgG
IgA
Scaled expression
min
max
UMAP-2
UMAP-1

Figure S7: Comprehensive phenotypic profiling of tissue B cell subsets by UMAP - related to Figure 6.

UMAP plot generated from an equal subsampling of tissues and then from that distribution, an equal subsampling of B cell subsets, using only phenotypic (not isotypic) molecules. Coordinates are identical to UMAP in Figure 6H.

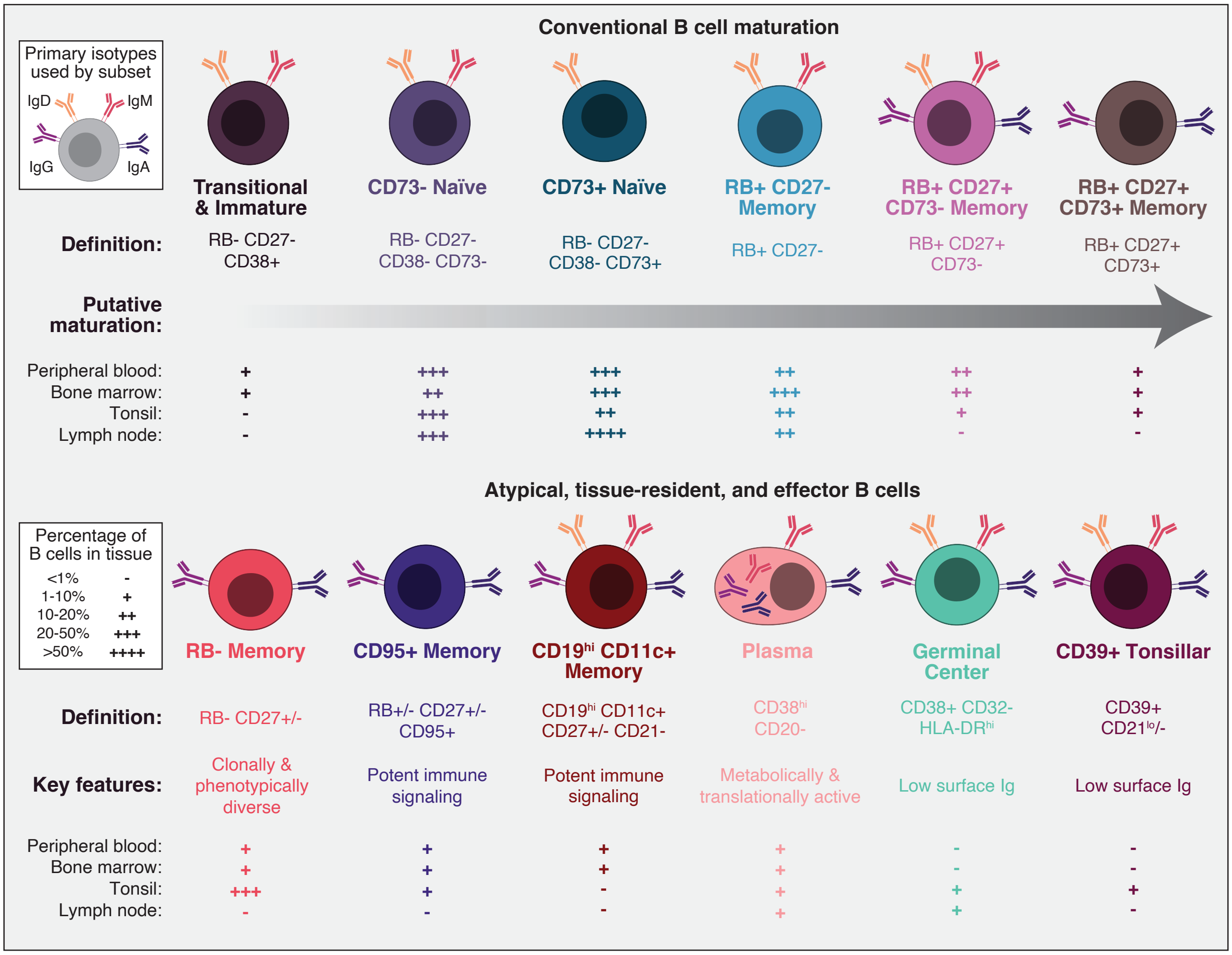
Conventional B cell maturation
Primary isotypes used by subset
IgD
IgM
IgG
IgA
Transitional & Immature
CD73- Naïve
CD73+ Naïve
RB+ CD27- Memory
RB+ CD27+ CD73- Memory
RB+ CD27+ CD73+ Memory
Definition:
RB- CD27- CD38+
RB- CD27- CD38- CD73-
RB- CD27- CD38- CD73+
RB+ CD27-
RB+ CD27+ CD73-
RB+ CD27+ CD73+
Putative maturation:
Peripheral blood: + +++ +++ ++ ++ +
Bone marrow: + ++ +++ +++ ++ +
Tonsil: - +++ ++ ++ + +
Lymph node: - +++ ++++ ++ - -
Atypical, tissue-resident, and effector B cells
Percentage of B cells in tissue
<1% -
1-10% +
10-20% ++
20-50% +++
>50% ++++
RB- Memory
CD95+ Memory
CD19hi CD11c+ Memory
Plasma
Germinal Center
CD39+ Tonsillar
Definition:
RB- CD27+/-
RB+/- CD27+/- CD95+
CD19hi CD11c+ CD27+/- CD21-
CD38hi CD20-
CD38+ CD32- HLA-DRhi
CD39+ CD21lo/-
Key features:
Clonally & phenotypically diverse
Potent immune signaling
Potent immune signaling
Metabolically & translationally active
Low surface Ig
Low surface Ig
Peripheral blood: + + + + - -
Bone marrow: + + + + - -
Tonsil: +++ + - + + +
Lymph node: - - - + + -

Figure S8: Summary of B cell subsets - related to Figure 4, 5, and 6.

Summary of features of B cell subsets described in this study. Cells are colored as in Figure 4, 5, and 6. Surface Ig illustrations relate the primary IgH isotypes used by each subset (see legend, upper left corner). Definition describes the key molecules that are uniquely expressed by each subset. Tissue diagram relates for each tissue, the percent of total B cells comprised by each subset (see legend, left middle). Putative maturation arrow (upper row) shows the proposed maturation ordering that occurs in the periphery. Key features (bottom row) describe unique features of each subset.
